# Gene-Specific Endothelial Programs Drive AVM Pathogenesis in *SMAD4* and *ALK1* Loss-of-Function

**DOI:** 10.1161/ATVBAHA.125.323908

**Published:** 2026-07-16

**Authors:** Olya Oppenheim, Wolfgang Giese, Hyojin Park, Elisabeth Baumann, Andranik Ivanov, Dieter Beule, Anne Eichmann, Holger Gerhardt

**Affiliations:** 1 Max Delbrück Center for Molecular Medicine in the Helmholtz Association, Berlin, Germany (O.O., W.G., E.B., H.G.).; 2 DZHK (German Center for Cardiovascular Research), Partnersite Berlin, Germany (O.O., W.G., E.B., H.G.).; 3 Charité Universitätsmedizin Berlin, Germany (O.O., E.B., A.I., D.B., H.G.).; 4 Berlin Institute of Health, Germany (A.I., D.B., H.G.).; 5 Cardiovascular Research Center, Department of Internal Medicine (A.E.), Yale University School of Medicine, New Haven, CT.; 6 Department of Molecular and Cellular Physiology (H.P., A.E.), Yale University School of Medicine, New Haven, CT.; 7 PARCC, INSERM, Université de Paris, France (A.E.).

**Keywords:** cell polarity, endothelial cells, gene expression, precision medicine, retina

## Abstract

**BACKGROUND:**

Hereditary hemorrhagic telangiectasia is a genetic disorder caused by loss-of-function mutations in components of the bone morphogenetic protein signaling pathway, leading to arteriovenous malformations. Most prior work has treated BMP (bone morphogenetic protein) component depletion as mechanistically interchangeable, yet whether distinct genes converge on a shared mechanism remains unclear. We aimed to understand the molecular relationship between BMP signaling and endothelial flow response that leads to arteriovenous malformation formation.

**METHODS:**

We expose human endothelial monolayers treated with small interfering RNA against *SMAD4* or *ALK1* to laminar flow and analyze flow-responsive transcriptomics, flow-responsive BMP signaling activation dynamics, cell polarity, and morphology. We analyze the cell-autonomous and noncell-autonomous migration dynamics of endothelial cells treated with *siSMAD4* or *siALK1*. Using the postnatal mouse retina model, we study endothelial cell distribution changes over time in mosaic settings, and assess the remodeling capabilities of *Smad4*^iECKO^ or *Alk1*^iECKO^, relative to littermate controls.

**RESULTS:**

This study shows that depletion of *SMAD4* or *ALK1* leads to fundamentally distinct mechanisms of vascular malformation. *SMAD4* deficiency enhances endothelial responses to blood flow, including transcriptional activation and migration against flow, causing excessive capillary pruning and the development of single large shunts. In contrast, *ALK1* deficiency disrupts flow sensing, impairs cell polarization and migration, and promotes a persistent angiogenic state, resulting in dense, hypervascularized networks. RNA sequencing across static and flow conditions identifies both flow-dependent and flow-independent transcriptional changes, suggesting early defects in endothelial fate specification. Mosaic in vitro models show that mutant cells co-opt neighboring wild-type cells, while in vivo tracking confirms mutation-specific migration behavior.

**CONCLUSIONS:**

These findings reveal divergent cellular programs driving arteriovenous malformations and underscore the need for gene-specific diagnostic and therapeutic strategies.

**What Are the Clinical Implications?**

This study identifies gene-specific mechanisms of arteriovenous malformation formation in endothelial cells with *SMAD4* or *ALK1* loss-of-function, 2 major mutations underlying hereditary hemorrhagic telangiectasia. Using multiple side-by-side analyses, the study reveals that *SMAD4* deficiency enhances flow-mediated remodeling, while *ALK1* deficiency impairs flow sensing and promotes angiogenic persistence. RNA sequencing revealed that endothelial gene expression varies across temporal and flow dimensions, with some programs emerging independently of flow and others shaped by hemodynamic forces, highlighting early divergence in cell fate and mechanosensory responses. Mosaic culture and retinal models demonstrate that mutant cells exert distinct non-cell-autonomous effects on wild-type endothelial cells. This work challenges the prevailing assumption of a unified pathogenic pathway in hereditary hemorrhagic telangiectasia and supports a model of gene-specific arteriovenous malformation pathogenesis, with distinct cellular and molecular drivers in *SMAD4*- versus *ALK1* loss-of-function. The findings provide a mechanistic basis for the variable clinical presentation observed in patients with different hereditary hemorrhagic telangiectasia mutations. Identification of gene-specific transcriptional signatures, such as persistent *ANGPT2* in *ALK1* loss-of-function or altered flow-response programs in *SMAD4* loss-of-function, may offer new biomarkers or therapeutic targets for early intervention before arteriovenous malformations become clinically symptomatic. Together, these results support a shift toward personalized therapeutic strategies, which could reduce reliance on invasive treatments and shift toward mechanism-based, precision medicine approaches in hereditary hemorrhagic telangiectasia and related vascular disorders.

The vascular system develops and remodels through a series of complex cellular processes that continuously adapt its shape and function to the local needs of tissues and organs.^1–6^ Defects in the fine-tuned coordination of endothelial cell (EC) collective migration, proliferation, perfusion-mediated stabilization, and cellular rearrangements in response to fluid shear stress (FSS) can lead to vascular malformations that can severely threaten organ function, or cause bleedings and life-threatening complications. Understanding the precise molecular and cellular mechanisms that drive aberrant vessel formation is fundamental to the development of precision therapy.

**See accompanying editorial by Baeyens**

Central to such understanding is the behavior of ECs during the formation and remodeling of the vascular network. Exactly how the collective behavior of ECs leads to the formation of a vascular network with hierarchical organization of arteries, capillaries, and veins remains incompletely understood. Whereas the initial vasculogenic assembly of vessels^2,5^ and subsequent sprouting angiogenesis^3^ are largely independent of blood flow, the stabilization of nascent vessels and the orderly pruning process that leads to the mature network depend on both the biomechanical forces of blood flow and related biochemical signaling events.^1,7–14^ Recent work identified flow-migration coupling as the orderly migratory response of ECs to changing FSS, which drives functional remodeling. Failure of ECs to engage in this flow-migration coupling process has been shown to occur in animal models of hereditary hemorrhagic telangiectasia (HHT).^7,9,10,15–18^

HHT is an autosomal dominant vascular disorder,^19^ and is characterized by the development of arteriovenous malformations (AVMs). Such malformations can occur in many organs and can be small (dilated small blood vessels called telangiectasias, typically appearing on the skin and nasal-mouth mucosa) or large intraorgan AVMs.^19–21^

The major genetic effectors causing this disorder are part of the BMP (bone morphogenetic protein) signaling pathway.^22–25^ Loss-of-function mutations in the coreceptor Endoglin, the type I receptor ALK1 (activin receptor-like kinase 1), and downstream DNA-binding protein SMAD4 (mothers against decapentaplegic homolog 4) account for the majority of diagnosed cases, and lead to HHT1,^26,27^ HHT2,^28,29^ or juvenile polyposis combined with HHT (JP/HHT),^23,30^ respectively.

When circulating BMP ligands bind to the extracellular domain of the ALK1-ENG (endoglin) heterotetramer, a conformational change in the intracellular domains subsequently leads to phosphorylation of receptor SMADs (mothers against decapentaplegic homologs) 1/5/9. These phosphorylated SMADs then bind to SMAD4 and translocate to the nucleus, where this complex regulates target-gene expression. As homozygous loss-of-function mutations in any of the effectors are embryonic lethal, accumulation of somatic mutations in the healthy allele drives the loss-of-function phenotype, in a process called loss of heterozygosity.^24^ Shunts appear in a sporadic manner and do not affect the entire vascular network within a tissue. Moreover, different somatic mutations accumulate in different cells and vascular beds.^31^ For these shunts to start developing in a mature and quiescent network, a second hit vascular injury, which causes a reactivation of the vascular bed and the exit from a quiescent state, is usually required to occur.^31,32^ Current treatment options include mostly symptomatic and surgically invasive approaches, while mechanism-based drug options are limited.^19,33^

The unpredictable behavior, variable presentation, and limited therapeutic options of AVMs pose significant clinical challenges, underscoring the need to elucidate the underlying mechanisms. In our search for critical and common molecular mediators and cellular mechanisms of HHT, we find that loss of *SMAD4* or *ALK1* surprisingly shows very different effects and influences the EC response to FSS in opposite ways, ultimately leading to different AVM pathomechanisms. Loss of *SMAD4* enhances collective migration against flow, leading to hyperpruning of proximal capillaries, therefore precipitating a shunt. In contrast, *ALK1*-deficient cells show a delayed and reduced migration against flow, coupled with angiogenic persistence, causing AVMs to grow via hypopruning and enlargement of capillaries. These findings provide valuable insights into distinct pathomechanisms that lead to AVM formation, offering potential avenues for improved diagnostics and mechanism-based treatment options.

## Methods

### Data Availability

The authors declare that all supporting data are available within the article and Supplemental Material. The raw RNA-Seq reads are deposited in the GEO database under accession number GSE282952.

### Cell Culture Experiments

Human umbilical venous ECs (HUVECs, PromoCell) were expanded and used in passages 2 to 4. Culture flasks were coated with 0.2% gelatin before seeding. Cells were cultured in EGM2-Bulletkit (endothelial growth media 2; Lonza) or MV2 (microvascular media 2; PromoCell) in a 37 °C humidity incubator with 5% CO_2_. For small interfering RNA (siRNA) knockdown, cells were seeded in T25 flasks and transfected after 24 hours at 60% to 70% confluence for *siCTRL* (Qiagen AllStars negative control, 40pmol), *siSMAD4* (Qiagen, FlexiTube GeneSolution for *SMAD4*: SI03089527, SI03042508, SI00076041, and SI00076020; 10 pmol each) or *siALK1* (Qiagen, FlexiTube GeneSolution for *ALK1*: SI02659972 and SI02758392, 20 pmol each), using RNAiMax transfection reagent (Thermo Fisher) diluted in OptiMem media and added to antibiotic free EBM2 media (endothelial basal media 2; Lonza). After 5 hours, the transfection mix was aspirated and replaced with full media.

### End Point Flow Experiments

HUVECs were harvested 24 hours after siRNA knockdown and seeded onto gelatinized 0.4 Luer ibiTreat slides (Ibidi) at a concentration of 2 million cells per ml and placed overnight in a CO_2_ incubator. The following day, slides were connected to perfusion units (Ibidi) and exposed to 0.6 Pa or 1.8 Pa shear stress for 4 hours. Cells that were exposed to shear stress for 16 hours were connected later on, and had a media exchange earlier that day. A static control for each condition was placed in the flow incubator for the same duration as the flow experiments. After each flow experiment, cells were either fixated with 4% paraformaldehyde (PFA) for subsequent immunofluorescence assays or lysed with RLT buffer (RNA lysis buffer for cells and tissues, RNAeasy RNA extraction kit, Qiagen) for RNA-based subsequent analysis.

### RNA Extraction for Quantitative Polymerase Chain Reaction, RNA Seq

After flow experiments, cells were lysed with 2 rounds of 200 μL RLT buffer and collected into tubes. Cell lysates were kept in −80 °C freezers until all relevant samples had been collected, then RNA extraction was completed with the RNAeasy kit according to the manufacturer’s instructions, including DNAse step. RNA concentration was assessed using NanoDrop. RNA was sent to the genomic core facility for RNA sequencing or used for quantitative polymerase chain reaction analysis. cDNA synthesis was performed using BioRad or Thermo Fisher cDNA kits according to the manufacturer’s instructions. Quantitative polymerase chain reaction was performed using Taqman probes on a QuantStudio 6 quantitative polymerase chain reaction machine in 384-well plates.

### Bioinformatic Analysis

RNA-seq quality control was performed at both raw-read and postalignment levels. Raw sequencing read quality was assessed using FastQC (v0.11.9),^34^ and QC reports were aggregated using MultiQC (v1.14).^35^ Raw-read QC metrics included per-base sequence quality, per-sequence quality, GC-content distribution, sequence duplication levels, read length distribution, and overrepresented sequences/adapter content. In addition, postalignment QC metrics were evaluated using STAR (v2.7.3a),^36^ Samtools (v1.161.1),^37^ QualiMap (v2.2.2d),^38^ Picard (v2.27.5),^39^ RSeQC (v5.0.1),^40^ and featureCounts (v2.0.3).^41^ Across all samples, the read length was 100 bp, and the FastQC GC content was highly consistent, ranging from 50% to 51%. STAR uniquely mapped reads were consistently high, ranging from 91.6% to 93.8% across samples. Samtools mapping rates were also high, with 95.2% to 97.2% mapped reads and 95.2% to 97.2% properly paired reads. The fraction of ambiguously mapped reads was low, with MAPQ 0 reads ranging from 0.6% to 1.4%. Insert sizes were within the expected range, with median insert sizes of 218 to 254 bp. Feature assignment rates were 76.7% to 81.6% (intronic reads were not counted), and QualiMap 5′ to 3′ bias values were close to 1, ranging from 1.09 to 1.16, indicating no major gene-body coverage bias. No additional raw-read trimming or base-quality filtering was applied; no samples were excluded on the basis of sequencing quality, and downstream analyses were restricted to high-confidence alignments by using uniquely mapped reads only.

RNA-Seq reads were mapped to the human genome (GRCh38.p7) using STAR (v2.7.3a).^36^ The read count matrix was generated using FeatureCounts (v2.0.3)^41^ using Gencode version 25 (Ensembl 85) annotation and the following parameters -t exon -g gene_id -s 2 -p. Differential expression analysis was carried out using DESeq2 (version 1.38)^42^ using default parameters. Briefly, gene expression is modeled using a negative binomial generalized linear model, which accounts for overdispersion in count data and is commonly used for RNA-seq differential expression analysis. Statistical significance was assessed using Wald tests, and resulting *P* values were corrected for multiple testing using the Benjamini-Hochberg procedure to obtain adjusted *P* values. Only genes with at least 5 reads in at least 3 samples were considered for the analysis. Gene set enrichment analysis was carried out on a *P* value sorted list of genes using the CERNO test from the R tmod package (v0.50.13)^43^ using MsigDB (R msigdbr package v7.5.1) Biological Pathways GO collection.

### Immunofluorescence, Imaging of In Vitro Flow Samples

After fixation, slides were blocked and stained with primary antibodies against VEcadherin (goat, AF938, R&D Systems; 1:1000); GM130 (mouse, 610822, BD Bioscience; 1:500); pSMAD1/5/9 (rabbit, 13820S, Cell Signaling; 1:500); pSMAD2/3 (rabbit, 80427-2-RR, Proteintech; 1:500); or KLF4 (rabbit, HPA002926, Sigma Aldrich; 1:500). Secondary antibodies were used in 1:400 dilution: donkey anti-goat IgG 568 (A11057, Thermo Fisher); donkey anti-mouse IgG 488 (A21202, Thermo Fisher); donkey anti-rabbit IgG 647 (A31573, Thermo Fisher). Nuclei were stained using a 1:1000 DAPI solution. Mowiol mounting media was mixed with Dabco solution (Sigma) at a 10:1 ratio and applied to slides. Slides were imaged on the Zeiss 980 Confocal inverted microscope with a 20× air objective by taking 10 non overlapping z-stack images per slide.

### Image Analysis of In Vitro Data

Images were processed using sum intensity projection and were then analyzed using the Polarity-JaM toolbox and web application.^44^ The full code is available at https://github.com/polarityjam. Polarity analysis (for nuclei-Golgi polarity, cell orientation) was performed via acquisition of a polarity index, which is an indicator of the concentration of the circular distribution, its value (limited between 0 and 1) indicating the collective orientation strength of the monolayer.^45^ In addition, the signed polarity index (V-score) varies between −1 and 1 and indicates the strength of polarization with respect to a given direction of polarization.

Additional features were extracted through the web application https://application.posit.mdc-berlin.de/polarityjam/: Nuclear marker accumulation was analyzed via the ratio of the mean intensity of pSMAD1/5/9 or KLF4 in the nucleus and cytosol. Cell area was extracted in µm^2^. Cell elongation was extracted as the major/minor axis ratio. Cell orientation of each condition was extracted as V-score per image from the circular statistics file.

### Live Migration Under Flow

For live migration experiments, cells were prepared and seeded in the same conditions as described for the end point flow experiments. On the day of the experiment, seeding MV2 media was aspirated and replaced with CO_2_ independent media (Promocell basal media without phenol-red and without sodium bicarbonate, supplemented with MV2 supplement kit, B-glycerolphosphate at a final concentration of 4.32 μg/μL and sodium bicarbonate at a final concentration of 0.0075%) with addition of Spy505 (Spyrochrome) nuclear dye at 1:1000 dilution and incubated at 37 °C for 4 hours. Fluidic units were placed inside the incubation chamber of the microscope and warmed up before the connection of the slides. Slides were connected and placed on multi slide stage insert. Time-lapse imaging ran for 48 hours and acquired 3×3 tiles with 3 slice Z-stacks for each position, with a time interval of 7.5 minutes. Migration analysis was then done using the TrackMate feature^46^ on Fiji (ImageJ) with the StarDist segmentation tool.^47^ Tracks were analyzed using a Python based pipeline,^48^ using the following libraries: pandas, NumPy, matplotlib, seaborn, and statannot.

For mosaic migration experiments, before seeding into flow slides, cells were labeled with CellTracker Green or Red (Thermo Fisher) with a 1:5000 dilution (final concentration 2 nmol/L) in EBM2 media and incubated for 45 minutes at 37 °C, then washed 3× with full MV2 media and placed in the incubator for about 4 hours. Cells were then trypsinized and harvested, and seeded at the same concentration with a 1:1 ratio between green and red cells. Live imaging began the following day, 48 hours after siRNA treatment. Time-lapse imaging was configured in the same manner and ran for ≈2.5 days (500 cycles with 7.5-minute intervals). Subsequent analysis for each channel was done in the same manner as described in the previous paragraph.

### Statistical Analysis of In Vitro Experiments

Statistical analysis of PolarityJam features was performed using Prism 10.1.1 (GraphPad). Image-based data were collapsed into independent experimental means, followed by 2-way ANOVA with full model parameters. Columns were defined as the siRNA treatment, and rows were defined by the flow condition. Multiple comparisons were performed between cell means within each row and each column using Tukey correction. Raw end point data normality was assessed in GraphPad Prism using Shapiro-Wilk and Kolmogorov-Smirnov tests together with QQ plots. Additional model-based residual diagnostics were performed in Python using pandas and NumPy for data handling, statsmodels for fitting the 2-way linear models and extracting residuals, SciPy for statistical diagnostics, and Matplotlib for diagnostic plots. Residual normality was evaluated using statistical tests together with QQ plots of standardized residuals. Homogeneity of variance across treatment-condition groups was assessed using Brown-Forsythe/median-centered Levene tests, with Spearman correlation between fitted values and absolute residuals included as an additional heteroscedasticity diagnostic. Where residual diagnostics indicated deviations from model assumptions, log-transformed analyses were evaluated as sensitivity checks; primary analyses were retained on the original measurement scale for interpretability. Analysis plots were generated on Image-based data using Python with Matplotlib for visualization and NumPy/pandas for data handling. Boxplots display the distribution of raw image-level measurements (median, interquartile range, and whiskers extending to 1.5×interquartile range). Individual raw image-level measurements are overlaid as jittered points. Sample sizes are indicated in figure captions, and analysis scripts are accessible at https://github.com/gerhardt-lab/polarityjam-HHT.

Statistical analysis of migration experiments was performed using Prism 10.1.1 (GraphPad). Cumulative directional and overall displacement, and mean directional and overall velocity were calculated in 10-hour intervals and analyzed using 2-way ANOVA with matched row factor, full model parameters, and Geisser-Greenhouse correction. Columns were defined as the siRNA treatment, and rows were defined by the time interval. Multiple comparisons were performed between columns within each row with the Holm-Šídák multiple comparison test. Data and residual normality were assessed using Shapiro-Wilk and Kolmogorov-Smirnov tests together with QQ plots. Residual and heteroscedasticity plots were inspected to evaluate model assumptions. Sample sizes are indicated in the figure caption, and data are presented as mean±SEM.

### In Vivo Experiments

All animal experiments were performed under a protocol approved by the Institutional Animal Care and Use Committee of Yale University (no.2023-11406).

Seven to 8 weeks old *Acvrl1*^*f/f*^ or *Smad4* ^*f/f*^ mice with *Cdh5*-CreERT2 and *mTmG* mixed genetic background were intercrossed for in vivo experiments. *Cdh5*-CreERT2 *mTmG* pups were used as the control group for mosaic labeling, and CreERT2 negative littermates were used as controls for full knockout. Gene deletion was induced by intragastric injections with 0.75 μg Tamoxifen (Sigma, T5648; 2.5 mg mL^−1^) into P5 pups for mosaic knockout and labeling, and 100 μg Tamoxifen into pups at P4 (for Alk1 deletion) or P1-3 (for Smad4 deletion). For mosaic population analysis, retinae were collected on P8 and P15, and on P5-P6 for regression analysis.

### Immunostaining and Imaging of Mouse Retinae

Retinae were prefixed in 4% PFA for 8 minutes at room temperature. Retinae were dissected, blocked for 30 minutes at room temperature in blocking buffer (1% fetal bovine serum, 3% BSA, 0.5% Triton X-100, 0.01% Na deoxycholate, 0.02% Sodium Azide in PBS at pH 7.4), and then incubated with specific antibodies in blocking buffer overnight at 4 °C. The next day, retinae were washed and incubated with IB4 (isolectin B4) together with the corresponding secondary antibody overnight at 4 °C, then washed and postfixed with 0.1% PFA and mounted in fluorescent mounting medium (DAKO, United States). High-resolution pictures were acquired using a Leica SP8 confocal microscope with a Leica spectral detection system (Leica TCS SP8 detector) and the Leica application suite advanced fluorescence software. Primary antibodies: IB4 (no. 132450, 1:400; Life Technologies), GFP (green fluorescent protein) Polyclonal Antibody, Alexa Fluor 488 (no. A-21311, 1:1000; Invitrogen), CD31 (cluster of differentiation 31; 553370; 1:200; BD), Collagen IV antibody (2150-1470; 1:400; Bio-Rad). Secondary antibodies: Alexa Fluor 488 anti-Rat (A21208, 1:500; Invitrogen), Alexa Fluor 568 anti-Rabbit (A10042, 1:500; Invitrogen).

### Image Analysis of In Vivo Mosaic Experiments

Analysis of mosaic-labeled mouse retinae was done as previously described.^49,50^ Briefly, maximum projections of the IB4 channel were used to create drawn masks of the retina outline, veins, arteries, and optic nerve in Fiji.^51^ Maximum projections of the GFP channel were used to create GFP masks.

For every pixel in the GFP mask, 3 numbers were computed (using drawn masks as referential): (1) distance to the nearest vein (d_v_); (2) distance to the nearest artery (d_a_); and (3) radial distance to the optic nerve (d_r_). From these measures, the relative distances by ϕ_v−a_=d_v_/(d_v_+da) were obtained. The EC distribution was computed by performing the operation for x randomly selected GFP-positive pixels in each retina, which were used as a proxy for EC distribution. A kernel density estimation was used to approximate the underlying EC distribution in the 2-dimensional coordinate system spanned by ϕ_v−__a_ and d_r_. Computational analysis scripts are accessible at https://github.com/gerhardt-lab/retina-VACS-HHT.

### Image Analysis of In Vivo Remodeling Experiments

Analysis of mouse retinae used in remodeling analysis was done using the 3DVascNet software.^52^ Briefly, z-stacks of CD31 and ColIV (collagen type IV) channels were uploaded into the software, along with a resolution file depicting the micron/pixel ratio of each image. Channels of the same retina were segmented independently from each other. After segmentation, a region of interest (ROI) was selected for each image, generally encompassing 1 retina leaflet. ROIs were skeletonized in 3-dimensional using the generated 3-dimensional mask from the segmentation step. The software outputs feature quantifications, including ROI volume and number of branching points. Regression and anastomosis events for each retina were quantified using a composite of the CD31 and ColIV channels and within the borders of the analyzed ROI. A regression event was classified by an absence of the CD31 signal, while ColIV staining remained intact: Partial absence of CD31 indicated the vessel is in an intermediate regression stage; complete absence of CD31 while ColIV staining remained intact indicated the final regression stage of a vessel; complete absence of CD31 and partial disruption of ColIV staining indicated long regressed vasculature. An anastomosis event was classified by partial or complete absence of ColIV staining while CD31 staining was present. Regression/anastomosis frequency was quantified via the proportion of regression/anastomosis events per 100 branching points in the CD31 channel. Branching points and Vessel density ratios were quantified by dividing the outputs of the CD31 by ColIV channels, and subsequent normalization to the ROI volume ratio.

### Statistical Analysis of In Vivo Experiments

Computational and subsequent statistical analysis of mosaic retinae was performed in Python. GFP^+^ coordinates of each retina were processed using pandas and NumPy and binned into summary descriptors on both axes, including the 10th, 25th, mean, median, 75th, and 90th percentiles. Principal component analysis of the arteriovenous and radial summary variables was then used to visualize joint changes in GFP^+^ endothelial distribution across the retinal coordinate space. Because the AV and radial variables were measured on different scales, features were standardized before principal component analysis (PCA) using scikit-learn’s StandardScaler, and PCA was performed using scikit-learn’s PCA implementation. PCA plots were generated using Matplotlib and Seaborn. Group differences in combined PC space were assessed by PERMANOVA using scikit-bio, based on Euclidean distance matrices generated with SciPy. Homogeneity of multivariate dispersion was evaluated using PERMDISP. Individual principal components were subsequently analyzed by 2-way ANOVA with genotype and collection time as factors using statsmodels, to support axis-specific interpretation. Residual normality was assessed using Shapiro-Wilk and Kolmogorov-Smirnov tests together with QQ plots of standardized residuals, and homogeneity of variance was assessed using Brown-Forsythe/Levene tests and Spearman correlation of fitted values with absolute residuals. Where diagnostics gave mixed results, the corresponding PC-specific ANOVA results were interpreted cautiously and considered secondary to the multivariate PERMANOVA analysis. The number of samples/replicates is indicated in figure captions, and analysis scripts are available at https://github.com/gerhardt-lab/retina-VACS-HHT.

Statistical analysis of in vivo samples used for remodeling analysis was performed using Prism 10.1.1 (GraphPad). Regression and anastomosis frequency, branching point density, vessel density, and anastomosis/regression ratio were analyzed using a 2-way ANOVA test with full-model parameters. Columns were defined by genotype, and rows were defined by the collection time point. Multiple comparisons were performed between cell means within each row and each column using Tukey correction. Raw end point data normality was assessed in Prism using Shapiro-Wilk and Kolmogorov-Smirnov tests, together with QQ plots. Additional model-based residual diagnostics were performed in Python using pandas and NumPy for data handling, statsmodels for fitting the 2-way linear models and extracting residuals, and Matplotlib for diagnostic plots. Residual normality was evaluated using Shapiro-Wilk and Kolmogorov-Smirnov tests together with QQ plots of standardized residuals. Homogeneity of variance across genotype-time-point groups was assessed using Brown-Forsythe/median-centered Levene tests, with Spearman correlation between fitted values and absolute residuals included as an additional heteroscedasticity diagnostic. Where residual diagnostics indicated deviations from model assumptions, log-transformed analyses were evaluated as sensitivity checks; primary analyses were retained on the original measurement scale for interpretability, and results were interpreted cautiously in relation to the plotted sample-level distributions. Number of samples/replicates is indicated in the figure captions, and data are presented as mean±SEM. Analysis scripts are available at https://github.com/gerhardt-lab/Remodeling-HHT.

### Use of Large Language Models

Large language models, including ChatGPT (OpenAI), were used to assist with nonscientific aspects of manuscript preparation, such as rephrasing existing text for clarity and readability. Large language models were also used to support code development and modification for statistical data analysis. The large language model did not generate new data or suggest novel analytical approaches. All input data were derived from existing analyses, and the resulting code and outputs were reviewed and validated by the authors. The updated scripts are available at https://github.com/gerhardt-lab/.

## Results

### RNA Sequencing Unveils Distinct Transcriptional Regulation Associated With *ALK1* and *SMAD4* Loss-of-Function

**Figure 1.**
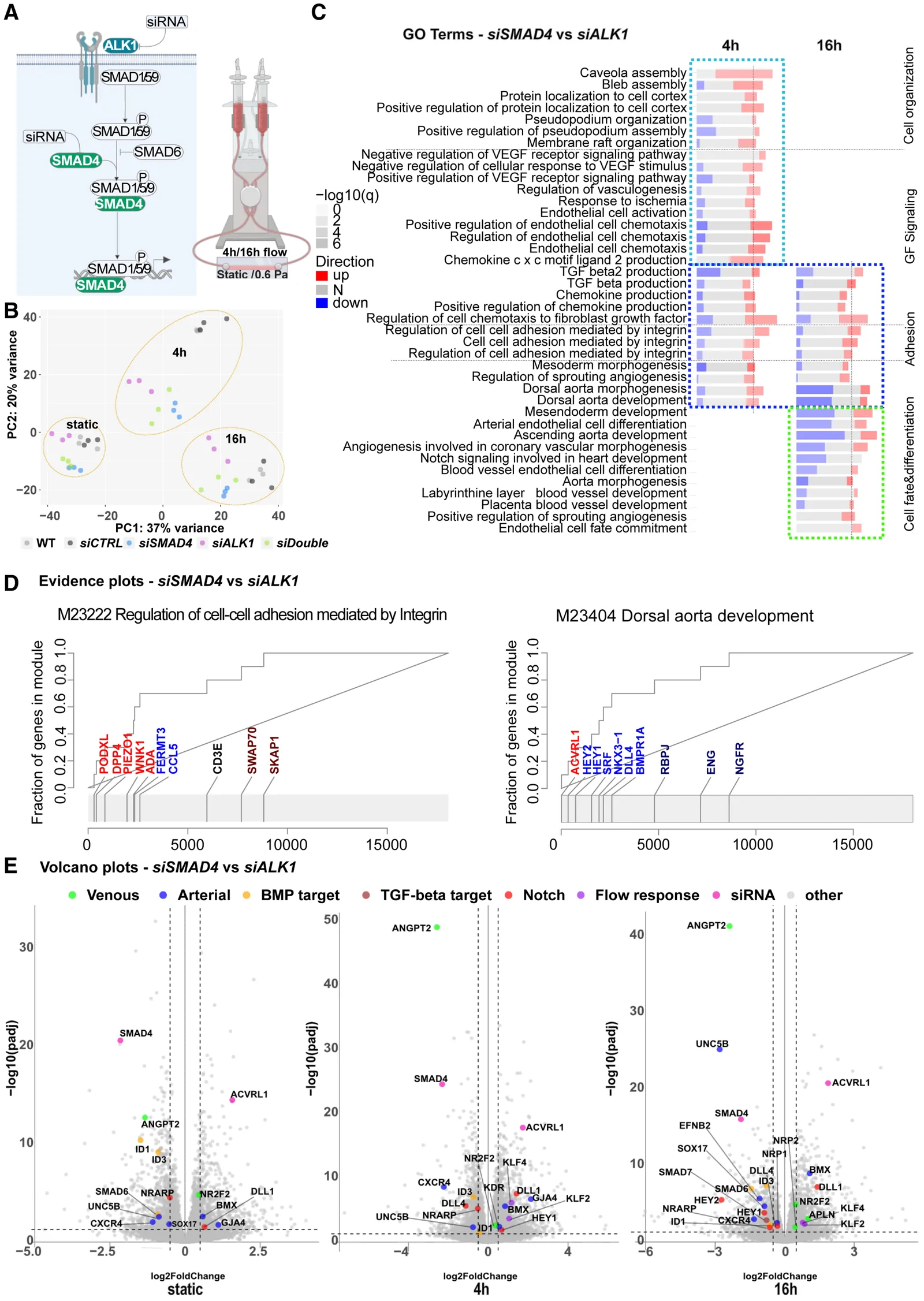
**Differential transcriptomic changes in endothelial cells (ECs) under flow. A**, Experimental schematic human umbilical vein endothelial cell (HUVECs) treated with siRNA against *ALK1* or *SMAD4* are exposed to 4 or 16 hours of laminar FSS 0.6 Pa (low shear stress [LSS] hereafter). **B**, Principal component analysis of RNAseq samples. Dashed circles group samples by flow duration. n=3 independent experiments. **C**, Panel plot of select biological processes, significantly (adjusted *P*<0.05) enriched in *siSMAD4*-treated samples vs *siALK1*-treated samples after 4 and 16 hours of LSS. The length of the horizontal rectangles represents the effect size of the biological process (area under the curve [AUC], see **D**). In red and blue are the fractions of significantly enriched, upregulated, or downregulated genes from that biological process. AUC_min_=0, AUC_max_=1. Vertical lines represent the threshold value (AUC=0.65) for each column. Dashed rectangles group GO-BPs according to their enrichment in specific time points. **D**, Evidence plots show GO-term enrichment across the ranked gene list (ordered by nominal *P* value) of 2 biological processes, with the step curve indicating the cumulative fraction of term genes captured; labels denote up- (red) or downregulated (blue) genes and are shaded by significance (bright=adj. *P*<0.05; dark=nominal *P*<0.05 only; black=not significant). **E**, Volcano plots of differentially expressed genes in *siSMAD4*-treated samples vs *siALK1*-treated samples, significance cutoff: Log2FC>0.5 or <−0.5. Select genes of interest are color-annotated. *SMAD4* and *ACVRL1* are annotated in magenta. ALK1 indicates activin receptor-like kinase 1; SMAD4, mothers against decapentaplegic homolog 4; and VEGF, vascular endothelial growth factor.

In search for common pathogenic downstream effectors of *ALK1* and *SMAD4* loss-of-function, we performed RNA sequencing on flow-mediated shear-stress exposed HUVECs following *SMAD4* or *ALK1* siRNA mediated knock-down (Figure S1.1). The FSS magnitude was adjusted to 0.6 Pa to represent the lower end of FSS values relevant to lesion-prone environments, as previous work has shown that AVMs in HHT models commence in capillaries and veins, but not in arteries.^22^ RNA samples were collected from HUVECs after 4 and 16 hours of flow exposure and from static controls (Figure 1A). PCA of all samples showed samples clustered primarily according to flow duration (Figure 1B), with untreated controls (light gray dots) and control siRNA-treated samples (dark gray dots, *siCTRL* hereafter) clustering together. Surprisingly, however, *ALK1* siRNA and *SMAD4* siRNA-treated samples did not cluster together. This is remarkable, given that *SMAD4* is a downstream component of the BMP9/10 signaling pathway triggered by the activation of *ALK1*. Instead, *siALK1*-treated samples (magenta dots) clustered separately from *siSMAD4*-treated samples (blue dots), and samples treated with both *siSMAD4* and *siALK1* (siDouble hereafter, green dots), clustered between the 2 single treatments in all time points. We therefore set out to investigate the differences in gene regulation in more detail.

Gene set enrichment analysis using Gene Ontology biological processes, directly comparing *siSMAD4*-treated samples versus *siALK1*-treated samples, revealed 6 clusters, based on flow dependence and duration (Figure S1.2, Data Set 1). Flow-dependent Gene Ontology biological processes (Figure 1C) were divided into 3 clusters: differential expression at early flow exposure (light blue), flow-dependent differential expression (dark blue), and differential expression at late flow exposure (light green). Interestingly, the early and late flow response Gene Ontology biological processes were quite different, with early flow exposure showing an enrichment in upregulation of biological processes associated with cell compartment organization, chemotaxis, VEGF (vascular endothelial growth factor) and TGF-beta (transforming growth factor beta) signaling in *siSMAD4*-treated samples, whereas late flow exposure exhibited enrichment in downregulation of Gene Ontology biological processes associated with cell fate and arterial differentiation in *siSMAD4*-treated samples, compared with *siALK1*-treated samples.

Evidence plots and volcano plots from the early flow response as well as late flow response exposed many differentially expressed genes, among which were canonical BMP targets (*ID1*, *ID3*, *SMAD6*); flow response genes (*KLF2/4*); Notch signaling associated genes (eg, *DLL1/4*, *HEY1/2*); arteriovenous identity associated genes (eg, *NR2F2*, *GJA4*, *NRP1/2*; Figure 1D and 1E; Data Set 2). Further direct examination of individual genes in comparison to controls suggested *ALK1* and *SMAD4* loss-of-function leads to highly differential effects on the endothelial response to flow (Figure S1.3).

### ALK1 Is Crucial for Flow-Induced BMP Pathway Activation, While SMAD4 Governs Its Duration

**Figure 2.**
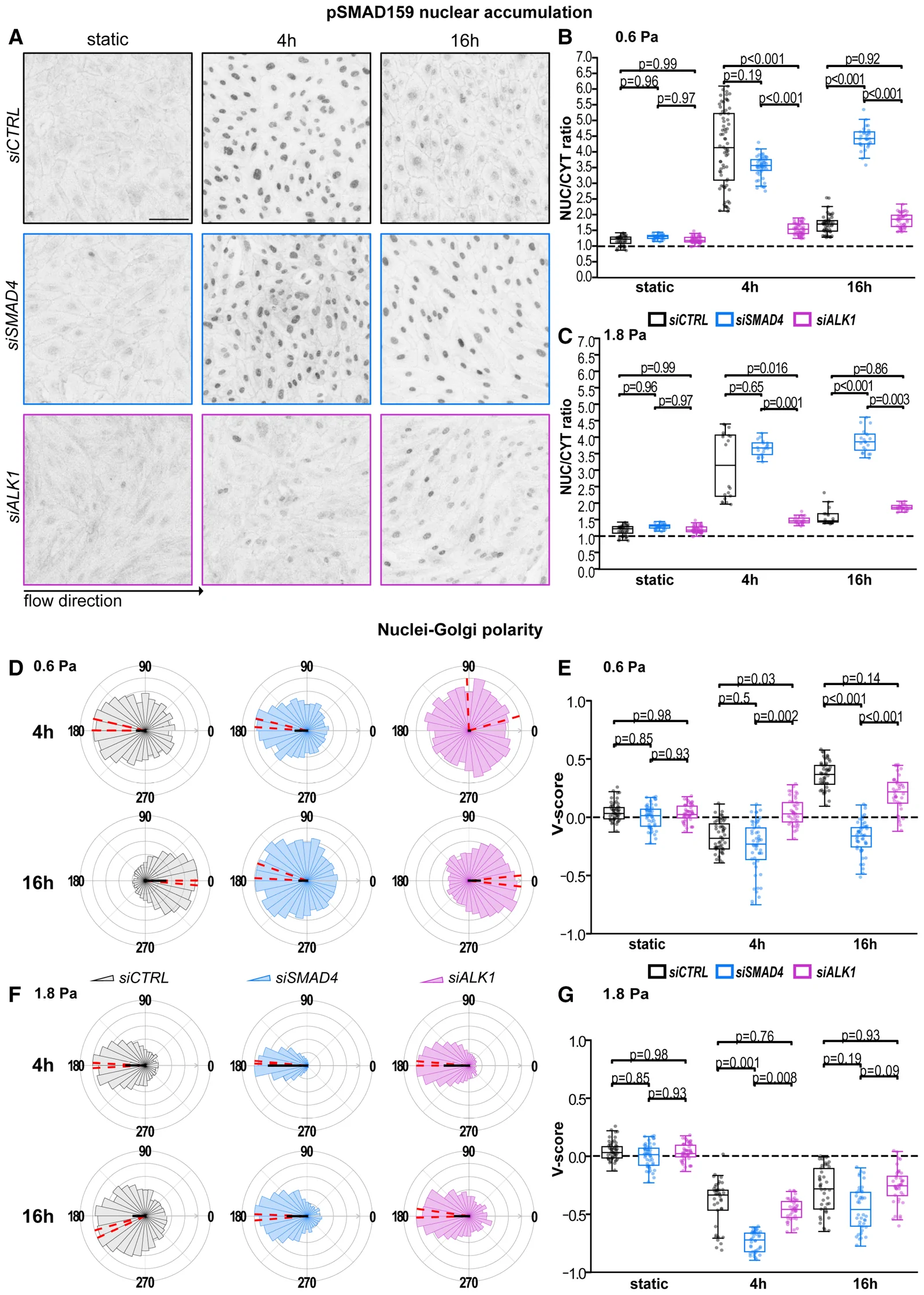
**BMP (bone morphogenetic protein) pathway activation and cell polarity under flow. A**, Representative pSMAD1/5/9 immunofluorescence images of siRNA-treated human umbilical vein endothelial cells (HUVECs) after exposure to 0.6 Pa (low shear stress [LSS]). Flow direction marked with an arrow. *siCTRL* annotated in black; *siSMAD4* annotated in blue; *siALK1* annotated in magenta. Scale bar 100 µm. **B** and **C**, Quantification of nuclear pSMAD1/5/9 signal, normalized to cytosolic signal at 0.6 and 1.8 Pa, respectively. Box- and scatter plots display the distribution of raw image-level measurements (≤10 images per experiment per condition). Statistical analysis on experimental means was done using 2-way ANOVA with Tukey multiple comparison correction. Individual sample size: n_static=3–5; n_0.6_Pa_4 h=5–7; n_0.6_Pa_16 h=3–5; n_1.8_Pa=2 (both time points). Full statistical analysis outputs are available in 02_statistics.xlsx. **D** and **F**, Rose diagrams depicting the Nuclei-Golgi polarity of *siCTRL*-, *siSMAD4*-, and *siALK1*-treated ECs exposed to 0.6 Pa (LSS) or 1.8 Pa (HSS) for 4 and 16 hours. The black line in each rose diagram points towards the mean direction of polarization, and its length represents the signed polarity index (plotted as V-score), which is an indicator for the variance of the distribution; the dashed red lines show the 95% CI. **E** and **G**, Statistical analysis of V-score values at 0.6 Pa (e) and 1.8 Pa (g). Box- and scatter plots display the distribution of raw image-level measurements (≤10 images per experiment per condition). Statistical analysis on experimental means was done using 2-way ANOVA with Tukey multiple comparison correction. Individual sample size: n_static=5; n_0.6_Pa=4-5 (both time points); n_1.8_Pa=4 (both time points). Full statistical analysis outputs are available in 02_statistics.xlsx. NUC/Cyt indicates nucleus-to-cytosol ratio.

As *SMAD4* is a direct transcriptional regulator of the *ALK1*-activated BMP pathway but also the TGF-beta pathway, it was essential to understand the roles of both *ALK1* and *SMAD4* in flow-induced BMP pathway activation. To test this, we exposed HUVECs to low shear stress (LSS) of 0.6 Pa (LSS hereinafter) for 4 and 16 hours as in the RNAseq setup, and assessed BMP pathway activation via assessment of nuclear pSMAD1/5/9 accumulation (Figure 2A and 2B). In static conditions, HUVECs displayed basal nuclear pSMAD1/5/9 levels. Exposing HUVECs to 4 hours of LSS showed increased and variable activation of the BMP pathway, which, however, subsided after 16 hours in the *siCTRL*-treated samples. The same flow regime in *siSMAD4*-treated samples led to a more uniform nuclear accumulation of pSMAD1/5/9 that remained active at 16 hours, while in *siALK1*-treated samples, no flow-induced activation of the BMP pathway was observed. Similar outputs were obtained for high shear stress (HSS) levels (1.8 Pa, HSS hereinafter, Figure 2C; Figure S2A). Because SMAD4 also mediates transcription downstream of TGF-beta signaling, we next tested whether the distinct phenotypes of *siSMAD4*- and *siALK1*-treated cells could be explained by differential engagement of the pSMAD2/3 pathway (Figure S2B and S2C). Quantification of nuclear accumulation by nuclear-to-cytosolic ratio did not reveal significant differences between static conditions. Upon flow exposure, changes in nuclear pSMAD2/3 accumulation were modest and clearly less pronounced than those observed for pSMAD1/5/9. To further examine whether transcriptional differences between *siSMAD4*- and *siALK1*-treated samples could be attributed to this pathway, we interrogated the RNA-seq data for TGF-beta/pSMAD2/3 target genes and found no substantial differential regulation apart from *SNAI1/2* and *COL3A1* (Figure S2D). Taken together with observed downregulation of canonical BMP pathway targets, these data suggest that ALK1 is essential for flow-induced activation of the BMP pathway, while SMAD4 absence results in a prolonged flow-dependent nuclear pSMAD1/5/9 accumulation that is uncoupled from canonical BMP transcriptional output. The comparatively minor effects on pSMAD2/3 activity and target-gene expression argue that this divergence is not primarily due to compensatory TGF-β/SMAD2/3 signaling.

### SMAD4 and ALK1 Deficiency Differentially Impact Cell Polarity in ECs

Cell polarity is a crucial aspect of development that is often characterized by the asymmetrical distribution of organelles in response to extracellular stimuli, such as biophysical forces.^53^

ECs exhibit a striking ability to establish flow-directed polarity, characterized by the positioning of the Golgi apparatus against the direction of blood flow, during their migratory response.^7,9,53^ There is growing evidence for changes in this front-rear polarity in AVMs, as well as in ECs depleted of BMP pathway components.^22,25^ To characterize these changes, we analyzed the nuclei-Golgi polarity of HUVECs after exposure to LSS and HSS for 4 and 16 hours (Figure 2D through 2G). In LSS conditions, we observed a weak polarization of *siCTRL*-treated samples against flow direction at 4h which reversed and intensified at 16 hours (Figure 2D through 2E). Surprisingly, *siSMAD4*-treated samples polarized against the flow direction in both time points, whereas *siALK1*-treated samples exhibited random polarization at 4 hours, followed by a weak polarization with flow at 16 hours. When exposed to HSS, cells polarized against flow in all treatments, although with the strongest effects in *siSMAD4*-treated samples (Figure 2F and 2G). The fact that even *siALK1*-treated samples polarize against flow, although they do not show induction of pSMAD1/5/9, suggests that polarization induced by HSS can at least be partially uncoupled from canonical *ALK1*-*SMAD4* signaling.^54^ In summary, our data show that ECs lacking *SMAD4* or *ALK1* respond very differently to FSS, particularly under LSS levels.

### FSS-Dependent Changes in Cellular Morphology Are Enhanced in the Absence of *SMAD4*

**Figure 3.**
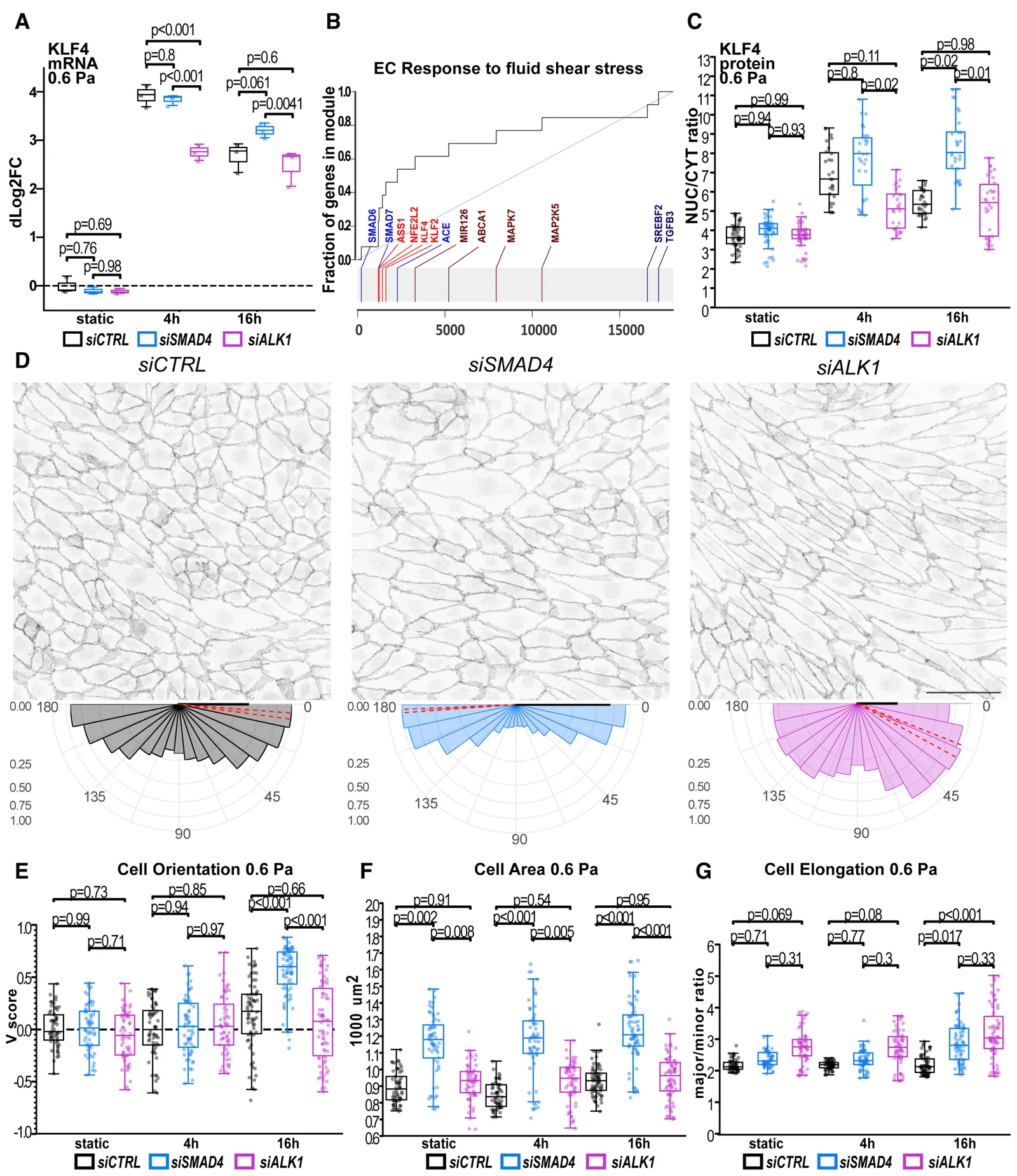
**Endothelial response to fluid shear stress (FSS) via KLF4 signaling and morphological changes. A**, Expression pattern of *KLF4* mRNA after exposure to 0.6 Pa (low shear stress [LSS]) in Log2FC scale, normalized to *siCTRL* static (black dashed line; RNA-seq data). Adjusted *P* values (padj) are indicated. n=3 independent experiments. **B**, Evidence plot of biological process “Response to fluid shear stress,” comparing *siSMAD4*-treated samples relative to *siALK1*-treated samples after 16 h of LSS. The step curve indicates the cumulative fraction of term genes captured; labels denote up- (red) or downregulated (blue) genes and are shaded by significance (bright=FDR-significant; dark=nominal *P*<0.05 only; black = not significant). **C**, Quantification of nuclear KLF4 signal at 0.6 Pa (LSS), normalized to cytosolic signal. Box- and scatter plots display the distribution of raw image-level measurements (≤10 images per experiment per condition). Statistical analysis on experimental means was done using 2-way ANOVA with Tukey multiple comparison correction. Sample size: n_static=5; n_0.6_Pa= 3 (both time points); n_1.8_Pa=2 (both time points). **D**, Representative immunofluorescence staining of VE-cadherin after 16 h of LSS (**top**) and the corresponding cell orientation diagrams (**bottom**). Scale bar 100 µm. **E** through **G**, Quantification of cell orientation V-score values (**E**), cell area (**F**), and cell elongation (**g**) at static, 4 h and 16 h of 0.6 Pa (LSS). Box- and scatter plots display the distribution of raw image-level measurements (≤10 images per experiment per condition). Statistical analysis on experimental means was done using 2-way ANOVA with Tukey multiple comparison correction. Sample size: n_static=6; n_0.6_Pa_4 h=6; n_0.6_Pa_16 h=7; n_1.8_Pa_4 h=4; n_1.8_Pa_16 h=4. Full statistical analysis outputs are available in 03_statistics.xlsx. EC indicates endothelial cell; KLF4, Krüppel-like factor 4; and NUC/Cyt, nucleus-to-cytosol ratio.

A key function of flow-migration coupling of ECs is the fine-tuning of vessel diameter. This fine-tuning depends on the ability of ECs to accurately sense flow and to undergo cellular rearrangements that affect their morphology, size, and orientation. Our data demonstrate that early during flow exposure, *siALK1*-treated samples fail to induce nuclear pSMAD1/5/9 accumulation and fail to polarize against flow during LSS exposure, suggesting they might have either lost the ability to sense LSS flow or to respond to it. The expression of KLF4 (Krüppel-like factor 4) transcription factor has been established as flow sensitive,^11,55,56^ as its mRNA expression increases in ECs on exposure to flow. Expression of *KLF4* mRNA in LSS samples confirmed that ECs differentially sense the exposure to LSS: *siCTRL*- and *siSMAD4*-treated samples showed elevated *KLF4* expression on LSS exposure, and also *siALK1*-treated samples showed elevated *KLF4* expression, albeit to a significantly lower magnitude (Figure 3A). The notion that lack of *ALK1* or *SMAD4* differentially affects transcriptional responses to flow is further supported by the evidence plot of the biological process “Response to fluid shear stress” (Figure 3B). Immunofluorescence staining for KLF4 protein also confirmed increased protein accumulation in the nucleus on exposure to LSS as well as HSS (Figure S3A). Albeit not to the same degree as seen at the RNA level, *siSMAD4*-treated samples had elevated nuclear KLF4 accumulation at both time points and at both LSS and HSS levels, whereas *siALK1*-treated samples had reduced nuclear accumulation only at LSS (Figure 3C; Figure S3A and S3B). In addition, we stained ECs for the adherens junction marker VE-Cadherin (vascular endothelial cadherin), allowing us to segment and analyze cell area, elongation, and orientation, and evaluate which of these are flow-dependent and dependent on the loss of *ALK1* or *SMAD4* (Figure 3D through 3G; Figure S3C through S3F). At both shear stress magnitudes, *siSMAD4*-treated ECs oriented themselves and elongated in a flow-dependent manner (Figure 3E and 3G; Figure S3D and S3F). Already in static conditions and throughout the flow experiment, *siALK1*-treated ECs in the monolayer formed distinct swirl-like clusters, where each sub-population maintained unique orientations independent of neighboring clusters (Figure S3C). In line with this qualitative phenotype and unlike *siSMAD4*-treated ECs, *siALK1*-treated ECs showed a tendency toward increased elongation already in static conditions, which became more pronounced under flow, particularly HSS. (Figure 3G; Figure S3C and S3F). After 16 hours of LSS, the cell area of *siALK1*-treated ECs remained similar to that of *siCTRL*-treated ECs (Figure 3F). In contrast, *siSMAD4*-treated ECs had a significantly larger cell area compared with *siCTRL*- and *siALK1*-treated ECs (Figure 3F; Figure S3E). Taken together, these observations further suggest an enhanced sensitivity to FSS in the absence of *SMAD4* and reduced sensitivity to LSS in the case of *ALK1* absence. In addition, both *SMAD4*- and *ALK1*-deficient ECs exhibit differential flow-independent morphological phenotypes.

### ECs Exhibit Distinct Migration Patterns in the Absence of ALK1 or SMAD4

**Figure 4.**
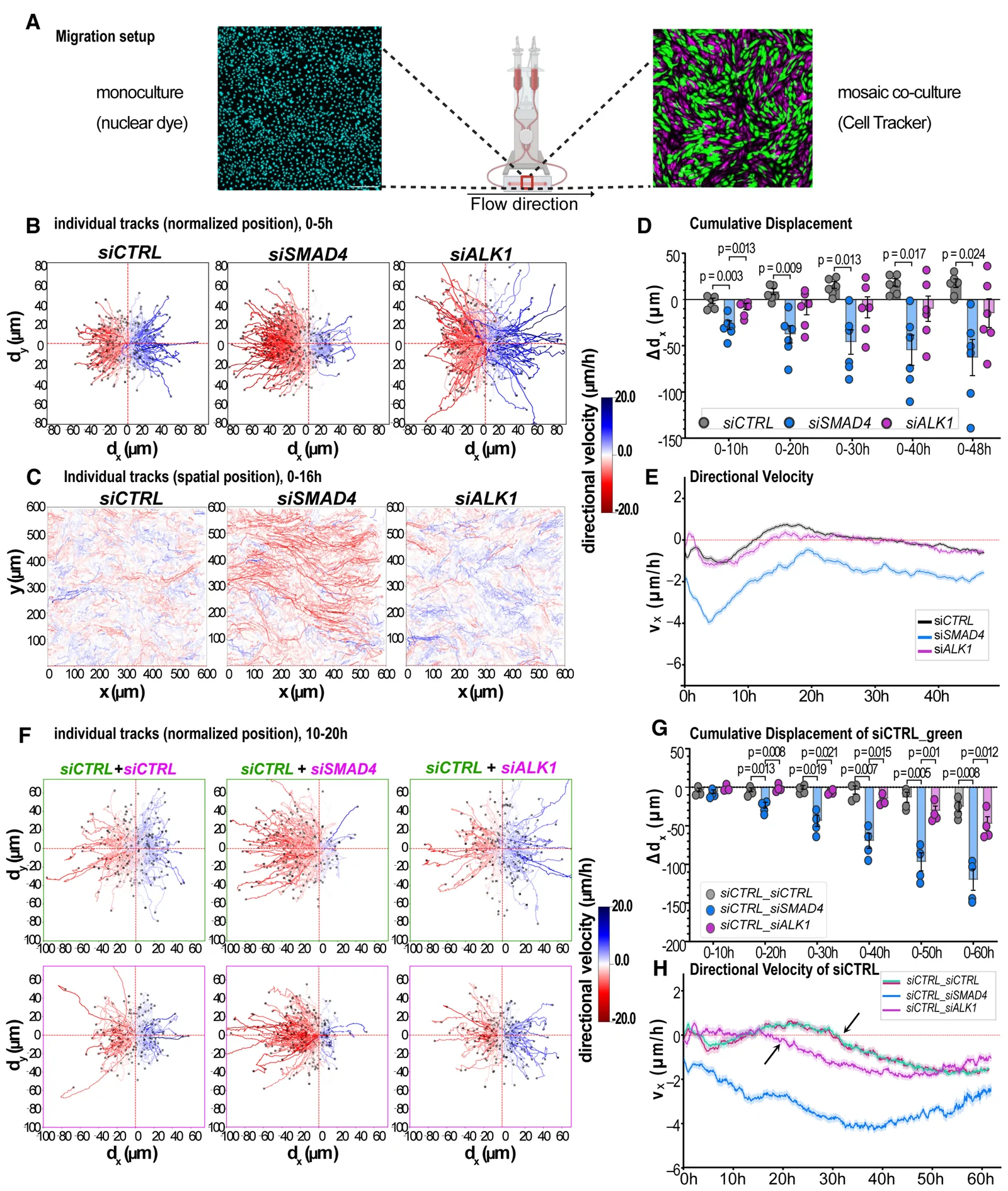
**Endothelial migration in vitro. A**, Live migration setup with representative endothelial cell (EC) monolayers before the onset of flow. Flow direction indicated by the arrow. EC monolayers are labeled with nuclear dye (monoculture) or CellTracker (mosaic coculture) before the flow experiment. The image field consists of 3×3 tiles. Scale bar 100 µm. **B**, Bootstrapped trajectory plots of initial 5 hours of migration for all conditions, 100 tracks per replicate, from n=6 independent experiments. The starting point of each cell trajectory has been normalized to start from (0, 0). The color intensity map represents the scale of parallel migration velocity with flow (blue) to against flow (red). **C**, Representative track images from individual samples of the initial 16 hours of migration. The spatial position of each track is kept. **D**, Cumulative displacement analysis parallel to flow. Data presented as mean±SEM. Statistical analysis was done using 2-way ANOVA with Holm-Šídák multiple comparison correction. **E**, Mean velocity of *siCTRL*-, *siSMAD4*-, or *siALK1*-treated ECs parallel to flow. n=6 individual experiments. **F**, Bootstrapped trajectory plots with normalized starting position of the *siCTRL*_green (**top**) and *siRNA*_red (**bottom**) cells in each coculture, from t=10 to t=20 hours. One-hundred tracks per replicate per color, from n=4 independent experiments. The color intensity map represents the scale of parallel migration velocity with flow (blue) to against flow (red). **G**, Cumulative displacement analysis parallel to the flow of *siCTRL*_green fraction. Data presented as mean±SEM. Statistical analysis was done using 2-way ANOVA with Holm-Šídák multiple comparison correction. **H**, Mean velocity of *siCTRL*-treated human umbilical vein endothelial cell (HUVECs) when cocultured either with themselves *(siCTRL_siCTRL*, green-red curve), or with *siSMAD4*- or *siALK1*-treated HUVECs (*siCTRL_siSMAD4*, blue curve and *siCTRL_siALK1*, magenta curve). Black arrows mark the beginning of migration velocity against flow for *siCTRL_siALK1* (≈20 h) and *siCTRL_siCTRL* (≈30 h).

The end point analyses of *SMAD4*- and *ALK1*-deficient ECs so far captured 2 contrasting phenotypes, suggestive of hypersensitivity and hyposensitivity to LSS, respectively. We, therefore, hypothesized that *siSMAD4*-treated ECs would migrate more efficiently against flow, whereas *siALK1*-treated ECs would fail to do so. To test this, EC nuclei were labeled and tracked over time under LSS, allowing analysis of both individual cell trajectories and collective migration behavior across siRNA conditions (Figure 4A through 4E; Figure S4A through S4C; Videos S1 through S3). Analysis of individual trajectories showed that, early in the experiment, *siCTRL*-treated ECs displayed a mild preference for migration against flow direction, with a substantial fraction of cells still moving with flow (Video S1; Figure 4B and 4C left panels). By comparison, *siSMAD4*-treated ECs (Video S2) exhibited a much more pronounced enrichment of trajectories directed against flow direction together with fewer perpendicular and downstream tracks, indicating a stronger directional response to LSS (Figure 4B and 4C middle panels). Although the fraction of cells moving with the flow was reduced, they moved with higher speed. *siALK1*-treated ECs (Video S3) also showed a robust increase in overall motility in all directions, indicating that the increased movement did not translate into efficient migration against flow (Figure 4B and 4C right panels). Cumulative displacement analysis along the flow axis confirmed these differences at the population level. *siSMAD4*-treated ECs showed a continuous net displacement against flow over the course of the experiment, whereas *siCTRL*- and *siALK1*-treated ECs displayed only a modest net shift within the monolayer (Figure 4D). Likewise, directional velocity analysis revealed that *siCTRL*-treated ECs initially migrated against flow, then transiently reversed direction, and ultimately slowed toward near-neutral net movement parallel to flow, consistent with a biphasic adaptive response (Figure 4E, black curve; Figure S4A). In contrast, *siSMAD4*-treated ECs accelerated more strongly against the flow direction and maintained migration against flow throughout the experiment (Figure 4E, blue curve; Figure S4A). *siALK1*-treated ECs behaved similarly to *siCTRL*-treated ECs, but their net directional response did not show a clear sustained upstream shift (Figure 4E, magenta curve; Figure S4A).

Although *siALK1*-treated ECs showed an early increase in overall motility, total migration velocity was broadly similar between all siRNA treatments (Figure S4B). In addition, *siSMAD4*-treated ECs displayed a cumulative total displacement advantage over much of the experiment (Figure S4C). Together, these findings support the idea that loss of *SMAD4* renders ECs hypersensitive to flow, leading to more persistent migration against flow, whereas the loss of *ALK1* reduces the efficiency with which ECs collectively orient their directional migration behavior, despite their high migratory capacity. *siCTRL*-treated ECs display an early response consistent with flow adaptation, suggesting that low shear stress elicits a slower adaptive remodeling response.

As HHT progresses by the loss of heterozygosity caused by somatic mutation of the remaining healthy BMP pathway alleles in only a subset of ECs within the vascular plexus, a mosaic setting is likely to more closely reflect the in vivo situation than uniform gene depletion. To model this scenario, we established mosaic cocultures in which control ECs were cultured together with either *SMAD4*- or *ALK1*-deficient cells, and the 2 populations were tracked separately under flow (Figure 4F through 4H, Figure S4D through S4I; Videos S4 through S6). Strikingly, mosaic conditions altered the migratory behavior of the co-cultured control cells. When *siCTRL*-treated cells were cultured together with *siSMAD4*-treated cells, the control population migrated more effectively against flow than in the *siCTRL_siCTRL* condition, as reflected by a strong increase in upstream-directed tracks, negative cumulative directional displacement, and directional velocity (Videos S4 and S5; Figure 4F through 4H, compare top-left and top-middle trajectory panels; Figure S4D and S4G). The opposite pattern was observed when *siCTRL*-treated cells were cultured with *siALK1*-treated cells. In this setting, *siCTRL*-treated cells exhibited a visible shift towards more positive migration velocity, with an increase in downstream-directed tracks and an average velocity close to 0 µm/hour during the first 20 hours (Videos S4 and S6; Figure 4F and 4H magenta; Figure S4D and S4G). Thus, the presence of *ALK1*-deficient cells initially impaired the ability of neighboring control cells to migrate efficiently against flow. Interestingly, this delay was transient, and *siCTRL*_*siALK1* eventually recovered and began migrating against the flow, ≈10 hours earlier than the *siCTRL_siCTRL* (Video S6; Figure 4H, black arrows; Figure S4D and S4G). Notably, the total migration velocity of *siCTRL* cells in the *siCTRL*_*siSMAD4* condition remained comparatively stable over time, whereas siCTRL cells in the *siCTRL*_*siCTRL* and *siCTRL*_*siALK1* conditions showed a modest decline during the course of the experiment (Figure S4E and S4H). Together, these data indicate that the consequences of *SMAD4* or *ALK1* loss are not purely cell-autonomous in mosaic settings. *SMAD4*-deficient cells promote a more strongly upstream-directed migration pattern not only in themselves but also in neighboring control cells, consistent with a non-cell-autonomous enhancement of flow-directed migration. Conversely, *ALK1*-deficient cells impose a transient non-cell-autonomous restraint on the upstream migration of adjacent control cells, in line with the reduced flow sensitivity observed cell-autonomously after *ALK1* loss. Overall, these findings suggest that both *SMAD4*- and *ALK1*-deficient ECs can influence the collective migratory behavior of surrounding wild-type cells under flow.

### Cells Lacking Alk1 or Smad4 Exhibit Distinct Population Movements In Vivo

**Figure 5.**
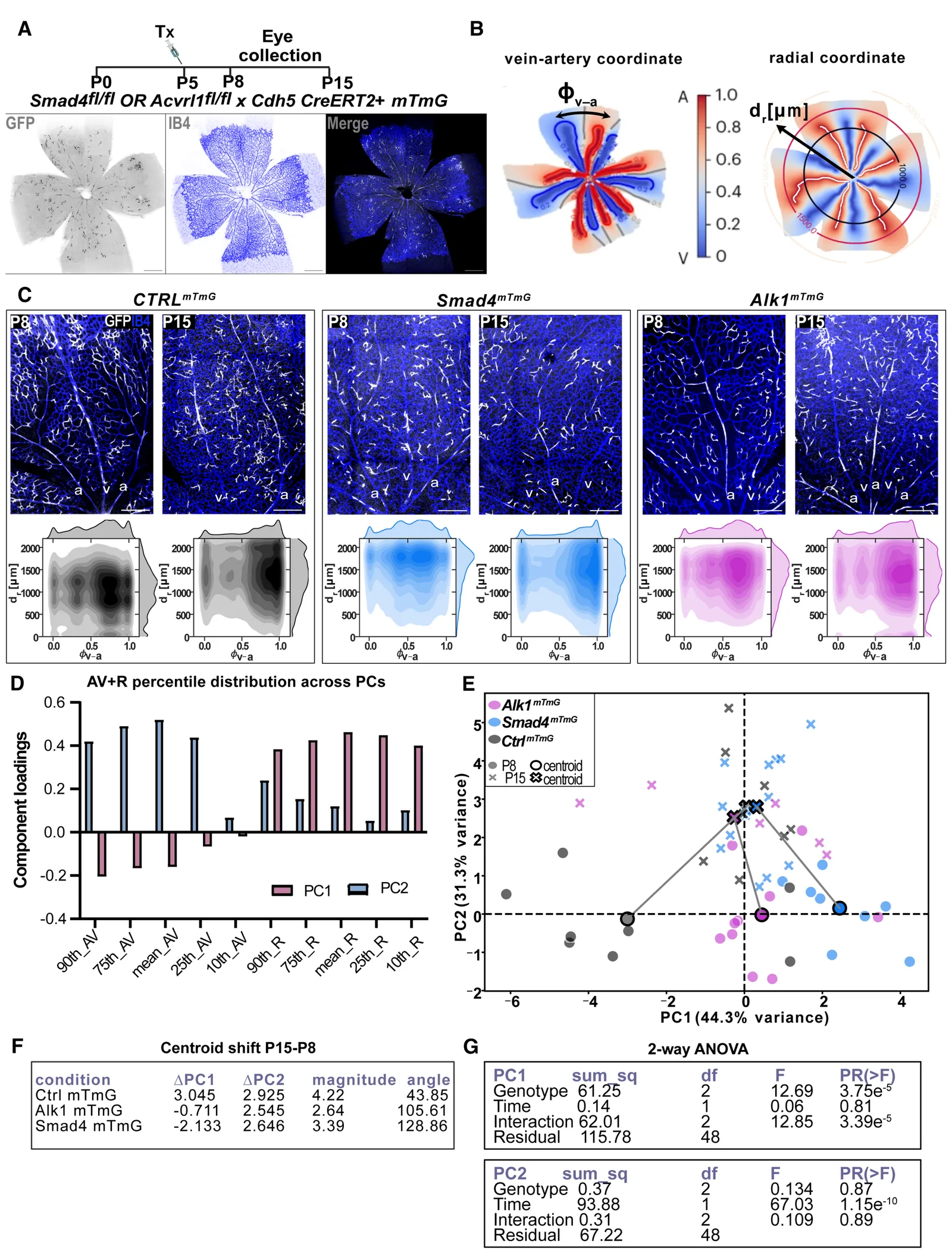
**In vivo endothelial cell (EC) population distribution analysis. A**, **Top**: Tamoxifen injection regimen. Single 0.75 μg injection of tamoxifen (T-OH) at P5 to induce mosaic gene deletion and subsequent expression of GFP, collection at P8 and P15. **Bottom**: Representative microscopy images of P8 retina: GFP, IB4, and merged channels. Scale bar: 500 µm. **B**, Schematic of vein-artery coordinate ϕ_v-a_ and radial coordinate d_r_ (µm). **C**, Representative images of retina sections at both time points for *CTRL*^mTmG^ (**left**), *Smad4*^mTmG^ (**middle**), and *Alk1*^mTmG^ (**right**) conditions; arteries, a; veins, v; scale bar, 250 µm. Resulting Kernel Density Estimate (KDE) plots for P8 and P15 for each condition on the bottom represent the GFP+ distribution across all retinae. Higher magnification images are available at Figure S5d. **D**. through **G**, Principal component analysis of AV+R axes. Component loadings (**D**) describe how each percentile in each coordinate contributes to PC1 (red) and PC2 (blue). PCA plot (**E**) is centered on the cross-genotype mean of P8. Circles represent P8 samples, Xs represent P15 samples. Bold circles/Xs represent the centroid of each genotype at P8 and P15, respectively. Gray arrows are calculated vector shifts from P8 to P15 centroids. **F**, Quantitative values of centroids’ shift on PC1 and PC2. **G**, Two-way ANOVA analysis output for PC1 and PC2. Additional statistical outputs are available in 05_statistics.xlsx. Number of retinae collected per condition: P8_*CTRL*^mTmG^=8; P15_*CTRL*^mTmG^=8; P8_*Smad4*^mTmG^=8; P15_*Smad4*^mTmG^=14; P8_*Alk1*^mTmG^=10; P15_*Alk1*^mTmG^=6.

Should the loss of *Alk1* or *Smad4* affect EC migration patterns in a similar way in vivo, we would predict that *Alk1* knock-out ECs show a mild inability to migrate against flow, whereas *Smad4* knock-out cells migrate even more effectively than control cells. To assess this possibility, we employed mosaic endothelial Cre-lox mediated genetic labeling in the postnatal mouse retina to follow population movements over time. *Cdh5*-CreERT2 *mTmG* (*CTRL*^*mTmG*^ hereinafter), *Smad4*^*fl/fl*^*-Cdh5*-*CreERT2 mTmG* (*Smad4*^mTmG^ hereinafter) or *Acvrl1*^*fl/fl*^*-Cdh5*-*CreERT2 mTmG* (*Alk1*^*mTmG*^ hereinafter) pups were treated with a low dose (0.75ug) of Tamoxifen at P5, to induce mosaic knockout of *Smad4* or *Alk1*, respectively; retinae were collected at P8 and P15 for IB4/GFP imaging (Figure 5A).

For each retina, GFP^+^ endothelial positions were mapped into a dual coordinate system consisting of the relative vein–artery coordinate (ϕ_v–a_) and the radial coordinate (d_r_[µm] – optic nerve to sprouting front), and visualized as 2-dimensional Kernel Density Estimate (KDE) maps (Figure 5B and 5C; Figure S5A).

Using this approach, we have previously shown that EC populations shift from the vein and plexus to the artery, as cells migrate against the direction of flow.^49,50^ Distance-to-artery analysis captured the GFP^+^ EC density within the artery and surrounding arterioles, which was reduced for *Smad4*^*mTmG*^ and *Alk1*^*mTmG*^ retinae at P8 relative to *CTRL*^*mTmG*^ (Figure S5B).

Between P8 and P15 *CTRL*^*mTmG*^ retinae showed a prominent shift of cells to the artery (position 1.0 on the ϕ_v–a_ axis, KDE plots at Figure 5C, left; Figure S5B and S5C gray curves). This shift was even more pronounced in the *Smad4*^*mTmG*^ retinae (Figure 5C, middle; Figure S5B and S5C blue curves), but was much less apparent in *Alk1*^*mTmG*^ retinae (Figure 5C, right; Figure S5B and S5C magenta curves).

To summarize these multidimensional redistribution patterns across the full per-retina distributions, we performed a PCA on combined AV and radial distribution summaries (Figure 5D through 5G). PC1, which explained 44.3% of the variance, loaded positively on radial summary measures and negatively on AV summary measures, thus capturing an opposing redistribution pattern of the GFP^+^ population (Figure 5D, red bars). Accordingly, within a given time point, more negative PC1 values indicate a relative enrichment of GFP^+^ ECs in the arterial side of the AV axis. PC2, which explained 31.3% of the variance, loaded positively across both AV and radial measures, consistent with a coordinated global shift in GFP^+^ EC population across the retinal map. At P8, both *Smad4*^*mTmG*^ and *Alk1*^*mTmG*^ retinas were positioned positively along PC1 relative to controls, consistent with a less artery-biased GFP^+^ EC distribution at this stage (Figure 5E, blue and magenta dots; Figure S5B, blue and magenta curves).

From P8 to P15, all genotype vectors showed a comparable positive shift along PC2, consistent with the overall developmental expansion of the retina (Figure 5E and 5F; gray arrows). In contrast, they diverged along PC1. Whereas *CTRL* ^*mTmG*^ samples shifted positively on PC1 (ΔPC1=3.045), *Smad4*^*mTmG*^ samples shifted negatively (ΔPC1=−2.13), indicating redistribution towards arterial structures (Figure 5E and 5F; Figure S5C, blue curves). By comparison, *Alk1* ^*mTmG*^ samples exhibited only a mild negative shift on PC1 (ΔPC1=−0.71), suggesting that their redistribution was driven predominantly by global radial expansion, with only limited redistribution towards the arteries (Figure 5E and 5F; Figure S5C, magenta curves).

PERMANOVA on PC1-PC2 detected significant group differences in the combined PC space (Figure S5D). Follow-up 2-way ANOVA on the individual PCA components showed that PC1 captured a significant genotype effect and genotype × time interaction, whereas PC2 showed only a significant time effect, consistent with a broadly shared P8-to-P15 developmental redistribution across genotypes along this axis (Figure 5G). Together, these results demonstrate that mosaic loss of Smad4 or Alk1 in ECs in vivo leads to differential distribution shifts that are consistent with our in vitro observations. Whereas the absence of Smad4 enhances redistribution toward arterial structures, the absence of Alk1 seems to impair this directional EC migration, but not completely prevent it.

### Loss of Smad4 Leads to Hyper-Pruning, While Loss of Alk1 Causes Hyper-Sprouting During AVM Formation In Vivo

**Figure 6.**
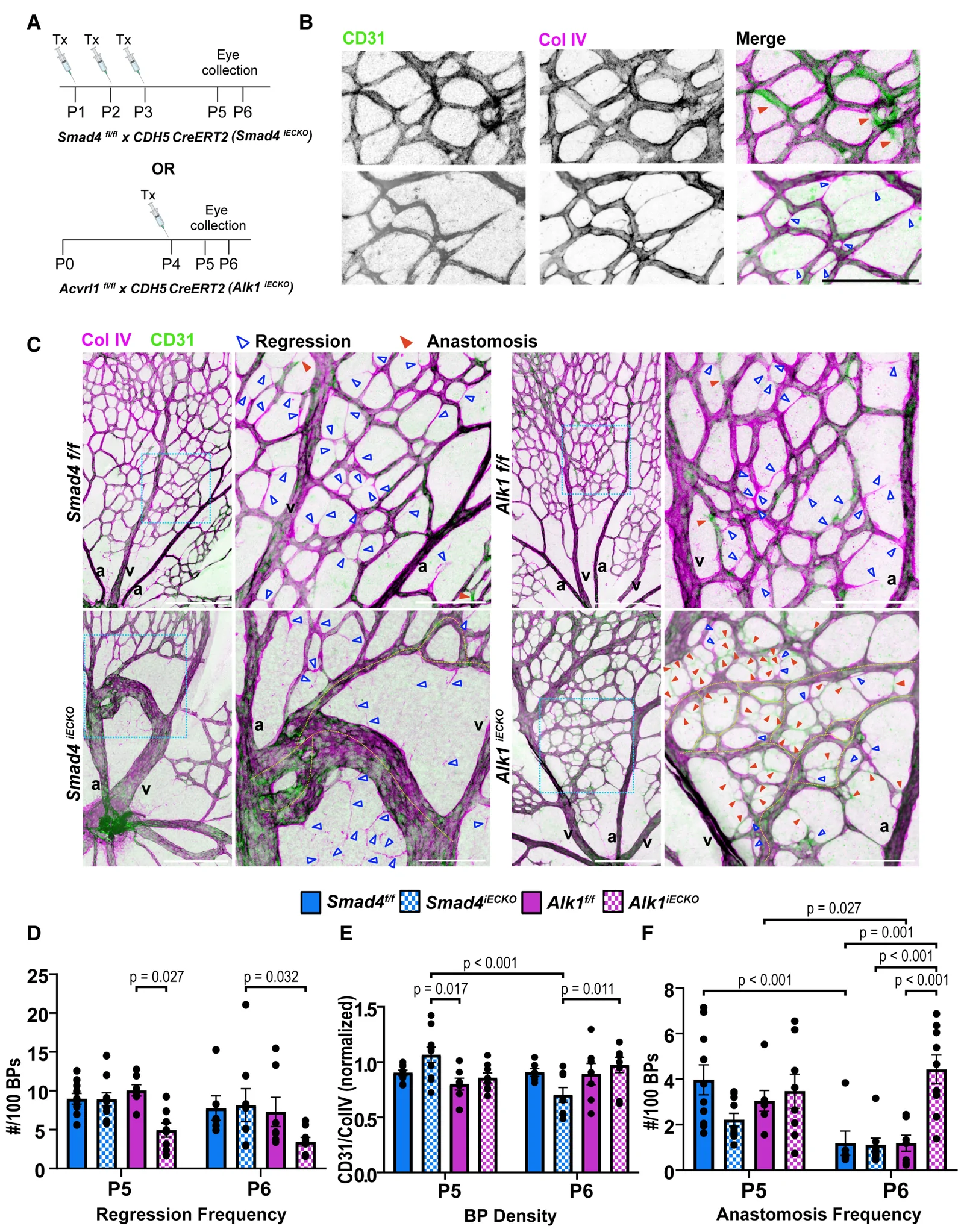
**In vivo regression analysis in postnatal retina. A**, Tamoxifen injection and sample collection regimen for *Smad4*^iECKO^ and *Alk1*^iECKO^, respectively. **B**, Representative grayscale and merge of maximum intensity projections of the CD31 (cluster of differentiation 31) channel and ColIV (collagen type IV) channel of *ALK1* littermate control retina. Orange triangles indicate anastomosis events, which are defined by partial or total absence of ColIV signal while the CD31 signal is present. Dark blue unfilled triangles indicate regression events, which are defined by the partial or total absence of CD31 signal within a vessel while ColIV signal is present. Scale bar 100 µm. **C**, Representative images of immunofluorescent staining of CD31 (green) and collagen type 4 (magenta) in P6 littermate controls (**top**) and mutated (**bottom**) retinae. Light blue rectangles annotate zoomed-in sections on the right. Dark blue unfilled triangles mark regression events, orange triangles mark anastomosis events. Arteriovenous malformations (AVMs) are highlighted in yellow dashed lines. Size marker 250 µm in main images, 100 µm in zoomed in images; v, vein; a, artery. **D** and **F**, Regression and anastomosis frequencies, presented by the number of regression and anastomosis events per 100 CD31+ branching points. The number of branching points is extracted from the 3DVascNet software for both CD31 and Col IV channels. Regression and anastomosis events are counted within a region of interest (ROI), which is also used to determine the number of branching points. Statistical analysis was done using 2-way ANOVA with Tukey multiple comparison correction. Data are represented as mean±SEM. **E**, Branching points’ density (no. branching points/ROI volume) ratios in littermate controls (full-color bars) and KO retinae (dotted bars). Statistical analysis was done using 2-way ANOVA with Tukey multiple comparison correction. Data are represented as mean±SEM. Full statistical outputs are available in 06_statistics.xlsx. Number of retinae collected per condition: P5_*Smad4*^fl/fl^=10; P6_*Smad4*^fl/fl^ =6; P5_*Smad4*^iECKO^=10; P6_*Smad4*^iECKO^=8; P5_*Alk1*^fl/fl^=7; P6_*Alk1*^fl/fl^=7; P5_*Alk1*^iECKO^=8; P6_*Alk1*^ECKO^=9.

Directional migration from vein to artery has been identified as the driving mechanism for pruning of the capillary plexus during vascular remodeling.^57,58^ We, therefore, hypothesized that the loss of *Smad4* should trigger hyper-pruning, hence increased capillary remodeling. In contrast, reduced sensitivity and propensity to migrate against flow, as seen in *ALK1*-deficient ECs, should rather trigger hypo-pruning, causing a hyperdense plexus. To analyze the remodeling states of the postnatal retinal vasculature of both *Smad4* knockout (*Smad4*^*iECKO*^ hereinafter) and *Alk1* knockout (*Alk1*^*iECKO*^ hereinafter), we labeled P5-P6 retinae using antibodies against CD31 (cluster of differentiation 31) and ColIV (collagen type 4) to visualize ECs and the vascular basement membrane, respectively (Figure 6A and 6B). Although *Smad4*^*iECKO*^ retinae overall showed a similar regression frequency to its littermate control (Figure 6C, left; Figure 6D, blue bars; and Figure S6A and S6B), we observed a decrease in the branching points density from P5 to P6 (Figure 6E, blue bars). In contrast, we observed a significant reduction in regression frequency in *Alk1*^*iECKO*^ retinae (Figure 6C, right; Figure 6D, magenta bars; and Figure S6C and S6D) in both time points, coupled with an increase in branching points density as well as vessel density (Figure 6E; Figure S6E). Vessel sprouting and anastomosis concentrated mainly near venous structures, sprouting>remodeling (S>R) transition zone,^7^ and sprouting front (Figure S6A through S6D). As expected, sprouting and anastomosis frequency were reduced over time in littermate controls and in *Smad4*^*iECKO*^ but were elevated in *Alk1*^*iECKO*^ retinae, particularly at P6 (Figure 6C, orange triangles, Figure 6F and Figure S6F). Furthermore, increased sprouting and anastomosis occurred in the remodeling/AVM zone of *Alk1*^*iECKO*^ retinae (Figure 6C, orange triangles and Figure S6D). When we established ratiometric analysis of anastomosis to regression as a measure of remodeling, *Smad4*^*iECKO*^ retinas showed a significantly reduced ratio at P5 compared to *Alk1*^*iECKO*^ retinae, which showed a strong increase at P5 and even more at P6 (Figure S6F). These results further support our hypothesis that *Smad4* and *Alk1* deletion cause opposing remodeling trajectories of the vascular plexus.

## Discussion

This study identifies distinct molecular and cellular mechanisms underlying AVM formation in ECs with *SMAD4* and *ALK1* loss-of-function. Our findings highlight that while both *SMAD4* and *ALK1* loss-of-function contribute to AVM pathogenesis, the underlying cellular behaviors and transcriptomic responses differ markedly, pointing to divergent mechanisms driving these vascular pathologies.

The results reveal that *SMAD4* loss-of-function enhances EC sensitivity to LSS, leading to increased hypertrophy and directional migration. The observed increase in *KLF4* expression on *SMAD4* deficiency confirms recent findings by Banerjee et al,^25^ lending strength to the concept that AVM formation is primarily driven by changes in EC mechanotransduction.^54^ The combined effects of increasing cell size, a phenotype first reported in *eng* mutants in zebrafish,^59^ and our unique observation of increased directional migration, orientation and polarization against flow, suggest an intriguing pathomechanism: this heightened migratory response accelerates vascular remodeling, resulting in premature capillary regression and the formation of large, high-flow AV shunts. Such a mechanism would explain the distinct appearance of AVMs in *SMAD4* mutant retinae. Our data provide several levels of evidence for increased flow sensitivity in *SMAD4*-deficient ECs: First, transcriptional changes highlight, besides classical flow-responsive genes like *KLF4* and *KLF2*, also prominent flow-regulated upregulation of cell surface proteoglycans involved in endothelial permeability regulation and vessel lumen regulation, like *PODXL*^60–63^ (Figure S1.3C). Second, flow-mediated cell shape changes and directional polarity are surprisingly enhanced rather than diminished in the absence of *SMAD4* (Figures 2 and 3). Third, directional EC population movements both in vitro (Figure 4) and in vivo (Figure 5) identify increased movement towards the artery, a behavior depending on flow-responses.^9^ Mosaic migration data further suggest that this heightened response is not confined to *SMAD4*-deficient cells themselves, but can bias the collective behavior of neighboring wild-type ECs. The cause for such enhanced flow sensitivity in *SMAD4*-deficient ECs remains to be identified. It should be noted that our work is not the first to identify heightened responses of ECs to flow when *SMAD4* is missing. Banerjee et al^25^ first reported a similar observation; however hypothesized this could indicate that altered flow-migration coupling, as proposed by Baeyens et al,^54^ may not be the driving cellular mechanisms of AVM formation. Moreover, Banerjee et al^25^ stated that distinct pathomechanisms driving AVM in *SMAD4* versus *ALK1* loss-of-function scenarios are unlikely. Our data instead identify distinct cell-autonomous gene-regulatory patterns, as well as highly distinct cellular behaviors in vitro and in vivo that point towards unique pathomechanisms.

One possibility is that *ALK1* and *SMAD4* differentially tune endothelial sensitivity to pro-angiogenic versus remodeling cues. Recent work by Franco et al identified competition between the chemotactic gradient of VEGF-A and the mechanical directional response to flow.^7^ Conceptually, this competition plays out in the capillary transition zone where VEGF-A gradients and flow gradients compete. Intriguingly, both processes involve VEGFR (VEGF receptor) signaling, with evidence for ligand-independent activation of VEGFR2 by flow, versus ligand-dependent activation in the context of sprouting. Furthermore, Notch signaling is linked to controlling arterial and venous cell fate acquisition,^64,65^ sprouting,^66^ as well as flow-induced remodeling.^67–69^ Thus, the loss of *ALK1* may drive hyper-sprouting and venous/capillary overgrowth by dysregulated Notch and increased ligand-dependent VEGFR2 signaling, whereas the loss of *SMAD4* selectively enhances Notch-related pruning through flow-dependent VEGFR2 mechanisms. In line with this idea, GO term analysis highlights a consistent pattern of transcriptional dysregulation in VEGFR signaling and integrin-mediated cell adhesion in *siSMAD4*-treated ECs relative to *siALK1*-treated ECs (Figure 1C and 1D; Figures S1.2 and S1.3): Gene sets involved in negative regulation of VEGFR signaling (M22989, M25085) show predominant upregulation of their constituent genes, while positive regulators (M15928) exhibit downregulation. In addition, GO terms related to integrin-mediated cell adhesion showed a predominance of upregulated genes (M23222, M23221, M13232). These data suggest a transcriptional reprogramming that may reduce VEGFR2 pathway activity, potentially altering cellular responses to flow stimuli. As VEGFR2 activation can occur via ligand-dependent (VEGF-A-mediated) or ligand-independent (flow-induced) mechanisms,^70^ shifts in the expression of key regulatory genes may influence the dominant signaling mode. In *siSMAD4*-treated cells, *DPP4* and *PODXL* were enriched as part of integrin-mediated cell adhesion GO Terms, and were uniquely upregulated: *DPP4* in a flow-independent manner, and *PODXL* in a flow-dependent manner, relative to both *siCTRL*- and *siALK1*-treated cells (Figure 1D; Figure S1.3C). Both genes are implicated in modulating endothelial adhesion and migration,^62,71,72^ and may contribute to the altered flow responsiveness observed. *DPP4* in particular has been linked to endothelial-to-mesenchymal transition through a TGF-β2-dependent, SMAD-independent mechanism.^73^ Its interaction with integrin β1 has been shown to upregulate VEGFR1 expression, which acts as a decoy receptor and suppresses VEGFR2 signaling. In parallel, we observed downregulation of *ITGAV* and *ITGB3*: integrins that facilitate VEGF-A binding and ligand-dependent VEGFR2 activation (Figure S1.3C).^74^ This combination suggests a shift away from VEGF-A-dependent signaling and toward ligand-independent, flow-induced VEGFR2 activation, which may underlie the enhanced sensitivity to flow observed in *SMAD4*-deficient ECs. However, further experimental validation is required to confirm these regulatory mechanisms and their functional consequences.

This molecular shift is mirrored in the striking vascular phenotype observed in vivo. *Smad4* loss-of-function in the postnatal mouse retina leads to large shunts devoid of a surrounding capillary plexus. Yet, even the peripheral plexus that will be hypoxic, showing upregulation of hypoxia-induced genes, like *ANGPT2*,^75^ shows no sign of hyper-sprouting, unlike in the case of *Alk1* mutant conditions. Thus, the lack of *Smad4* appears to selectively increase endothelial responses to flow, while reducing endothelial responses that are normally triggered by hypoxia. This phenotypic divergence reinforces the notion that *Smad4* plays a key role in balancing competing angiogenic cues and may act as a molecular switch between sprouting- and flow-mediated endothelial programs.

In contrast, *ALK1* loss-of-function appears to impair endothelial sensitivity to LSS, disrupting vascular remodeling and leading to reduced capillary regression (Figure 6). We observed a blunted upregulation of *KLF4* and diminished responses to LSS across multiple parameters, including reduced polarization, orientation (Figures 2 and 3), and migration of *ALK1*-deficient cells both in vitro and in vivo (Figures 4 and 5). This remodeling failure results in hypervascularized networks with dense, multicapillary AVMs (Figure 6C through 6F). Importantly, *ALK1*-deficient cells are not globally incapable of migration. Although directional migration is impaired, overall motility remains preserved, and collective migration can recover over time. In mosaic monolayers under flow, *siALK1* cells show reduced directional migration during the first half of the experiment; however, in the second half, both *siCTRL* and *siALK1* populations migrate against the flow direction. Similarly, in mosaic retinae, *Alk1*-deficient GFP^+^ ECs still redistribute toward the artery, albeit less efficiently. Thus, collective migration against the flow is impaired but not abolished. In a confluent endothelial monolayer, however, migration under flow is not purely cell-autonomous, but emerges from the integration of flow sensing, intercellular junctional coupling, and force transmission across neighboring cells.^76,77^ Within such a mechanically coupled sheet, wild-type ECs may therefore sustain or entrain directional movement of adjacent *ALK1*-deficient cells by imposing a collective balance of forces that still favors upstream rearrangement. In this scenario, the primary defect in *ALK1* loss-of-function would not be the inability to migrate per se, but a reduced capacity to initiate or reinforce flow-aligned collective behavior within the mechanically integrated monolayer. Together, these findings suggest that *ALK1* deficiency alters how flow-derived cues are interpreted and integrated at the population level, rather than eliminating the mechanical machinery required for migration.

However, our transcriptional data point to a more fundamental defect in endothelial fate specification and quiescence that arises even without, and thus independently of, flow onset. *ALK1*-deficient ECs show downregulation of key fate regulators, including the arterial markers *GJA4* (encoding Connexin37) and *CDKN1B* (encoding p27; Figure S1.3B),^64^ as well as the venous markers *BMP4*^8^ and *NR2F2* (encoding COUP-TFII [chicken ovalbumin upstream promoter-transcription factor II]; Figure S1.3D).^65,78^ In addition, Notch signaling appears uncoupled from flow stimuli: canonical Notch targets *DLL4*, *HEY1*, and *HEY2* do not respond appropriately under shear stress, particularly at 4 h (Figure S1.3B). These early changes coincide with persistent *ANGPT2* upregulation and downregulation of its receptor *TEK* (encoding Tie2 [tyrosine kinase with immunoglobulin and epidermal growth factor homology domains 2]; Figure S1.3D). Given that *ANGPT2* is a potent pro-angiogenic factor normally suppressed under flow to promote quiescence,^79,80^ this pattern suggests that *ALK1*-deficient ECs remain in an immature, angiogenic-like state and fail to transition into a stable, flow-responsive phenotype. This finding is also supported by recent work in *ALK1*-null hiPSC-derived ECs, where *ANGPT2* upregulation and reduced pSMAD1/5/9 signaling similarly marked a persistent angiogenic state.^81^

Consistent with this interpretation, and despite the inherent constraints imposed by differences in induction timing and AVM onset between *Alk1*^*iECKO*^ and *Smad4*^*iECKO*^ models, the most pronounced in vivo phenotype in *Alk1*^*iECKO*^ retinae was a marked increase in sprouting and anastomosis frequency, coupled with reduced regression (Figure 6; Figure S6). This hyper-angiogenic remodeling pattern was unique to *Alk1* deletion and was not observed in *Smad4* knockouts. These findings suggest that persistent angiogenic activation, rather than a primary defect in motility, is a dominant driver of AVM expansion in an *Alk1*-deficient endothelium.

Although previous studies comparing phenotypes and pathomechanisms of AVM formation between *SMAD4*, *ALK1*, and *ENG* depletion highlighted similarities, including upregulation of *ANGPT2*,^82^ we only find *ANGPT2* upregulation in *ALK1*-deficient cells, in line with the notion of failed induction of quiescence.^79,83^ A possible reason for this apparent discrepancy is the fact that we analyzed cell-autonomous gene regulation in ECs under flow, in the absence of pathology. Once an AVM has formed in vivo, studying altered gene regulation will report changes that may be primary, but also changes that are secondary to the altered tissue environment, including hypoxia. *ANGPT2* is a canonical marker of endothelial activation by hypoxia, but also in inflammatory conditions.^80,84^ The fact that large areas of the endothelium upregulate *Angpt2* in bulk RNAseq of isolated ECs from *Smad4* mutant retinae,^75^ like those from *Alk1* mutant retinae, is not necessarily evidence for a common causative *ANGPT2*-induced pathomechanism. Nevertheless, *ANGPT2* overexpression may contribute as a driver of wider tissue responses in both conditions.

Although AVMs are often regarded as high-flow vessels,^21,23,54^ several studies conclude that AVMs preferentially arise in regions of comparatively low flow, such as veins and microvasculature, rather than arteries.^22,85^ Our findings support this view by showing that the contrast between *siSMAD4*- and *siALK1*-treated ECs is most striking under LSS, whereas under HSS the responses become more similar across genotypes (Figures S2 and S3). Under these conditions, *siALK1*-treated ECs exhibit nuclear KLF4 accumulation comparable to controls and partially restored alignment parallel to flow, while *siSMAD4*-treated ECs still retain the strongest overall flow response. Together, these observations suggest that abnormal endothelial interpretation of LSS is sufficient to initiate maladaptive remodeling, whereas the high-flow state of mature AVMs is a consequence of shunt formation.

Our findings challenge the notion that AVM formation in *SMAD4* and *ALK1* loss-of-function situations is caused by a unified pathomechanism. Instead, they support a model in which *SMAD4* loss-of-function amplifies flow-driven vascular remodeling programs, whereas *ALK1* loss-of-function primarily destabilizes endothelial fate and maintains cells in a persistent angiogenic state. In this framework, *ALK1*-deficient endothelium remains capable of migration, particularly within a wild-type environment, but fails to properly couple flow sensing to quiescence and pruning. These insights align with prior studies implicating BMP signaling dysregulation in AVM pathogenesis^28,66,86^ and place BMP signaling as a central inter-pathway regulator of the shear-stress set point,^15^ along with Notch and VEGFR signaling.^66,68,87^ In this view, BMP-SMAD signaling does not simply respond to flow, but acts as a gain controller that determines how strongly a given shear magnitude is interpreted by the endothelium, thereby tuning the onset of flow-mediated polarization, migration, pruning, and stabilization.^25,87,88^ Our data align well with this set-point model, but suggest that *ALK1* and *SMAD4* tune the set point in distinct and even opposing ways.

This study opens several avenues for future research. The observed differences in transcriptional and behavioral responses between *SMAD4* and *ALK1* loss-of-function highlight the need for further mechanistic studies using multi-omics approaches. Combining transcriptomics, proteomics, and single-cell analyses across various tissue contexts could clarify which signaling effectors are primary drivers of AVM formation versus secondary responses to established pathology. Additionally, exploring factors such as ANGPT2 (angiopoietin 2) in *ALK1* loss-of-function and DPP4 (dipeptidyl peptidase-4) in *SMAD4* loss-of-function could uncover novel targets for modulating EC behavior.

In conclusion, this work provides a mechanistic framework for understanding AVM pathogenesis and underscores the need for pathway-specific therapeutic strategies in addressing vascular anomalies, such as HHT.

## ARTICLE INFORMATION

### Acknowledgments

The authors thank Leducq ATTRACT^16^ network members Hemaxi Narotamo and Claudio Franco, Douglas Marchuk, David Hardman, and Miguel O. Bernabeu for providing ongoing support throughout this project. The authors thank Tatiana Borodina of the Max-Delbrück-Center for Molecular Medicine in the Helmholtz Association (MDC)/Berlin Institute of Health (BIH) Genomics Technology Platform at the MDC and BIH, Berlin, Germany (https://www.mdc-berlin.de/de/forschung/infrastruktur/technologie-plattformen) as well as Eireen Bartels-Klein, Irene Hollfinger, and Jan-Phillip Albrecht for their technical assistance and guidance.

### Author Contributions

O. Oppenheim contributed to conceptualization, data curation, formal analysis, investigation, methodology, software, project administration, validation, visualization, writing the original draft, and writing-review and editing. W. Giese contributed to in vitro data formal analysis, methodology, software, visualization, and writing-review and editing. H. Park contributed to the in vivo data investigation, methodology, and writing the original draft. E. Baumann contributed to in vivo data formal analysis, methodology, software, and visualization. A. Ivanov contributed to transcriptomic data curation and formal analysis, methodology, investigation, and visualization. D. Beule contributed to data curation, formal analysis, methodology, software, visualization, and writing-review. A. Eichmann contributed to conceptualization, funding acquisition, investigation, and writing-review.

H. Gerhardt contributed to conceptualization, resources, funding acquisition, supervision, project administration, and writing-review and editing.

### Disclosures

This article includes results of the doctoral thesis entitled “Distinct endothelial pathomechanisms drive arteriovenous malformations in *ALK1* or *SMAD4* loss-of-function conditions” submitted by O. Oppenheim to the faculty of Charité Universitätsmedizin Berlin in 2025.^89^ The other authors report no conflicts.

### Supplemental Material

Figures S1–S6

Major Resources Table

ARRIVE guidelines

Analysis and Statistics Files

Videos S1–S6

## Supplementary Material
