# Supplementary material for: Gene-Specific Endothelial Programs Drive AVM Pathogenesis in SMAD4 and ALK1 Loss-of-Function
Source: Arterioscler Thromb Vasc Biol. 2026 Jul 16;46(9):e323908. doi: 10.1161/ATVBAHA.125.323908 (PMC13506206; doi:10.1161/ATVBAHA.125.323908)

Full unedited blots for Fig. S1.1

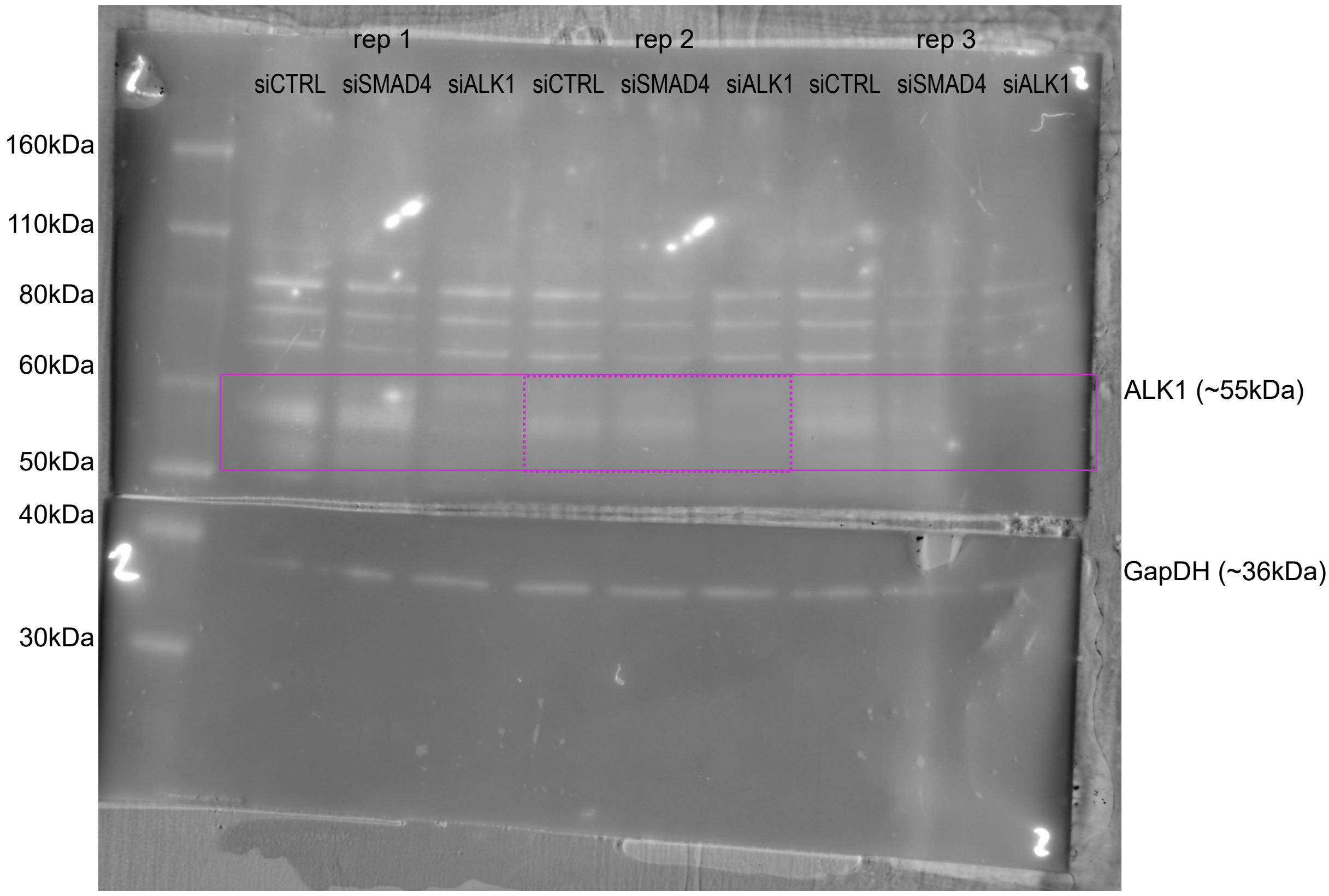

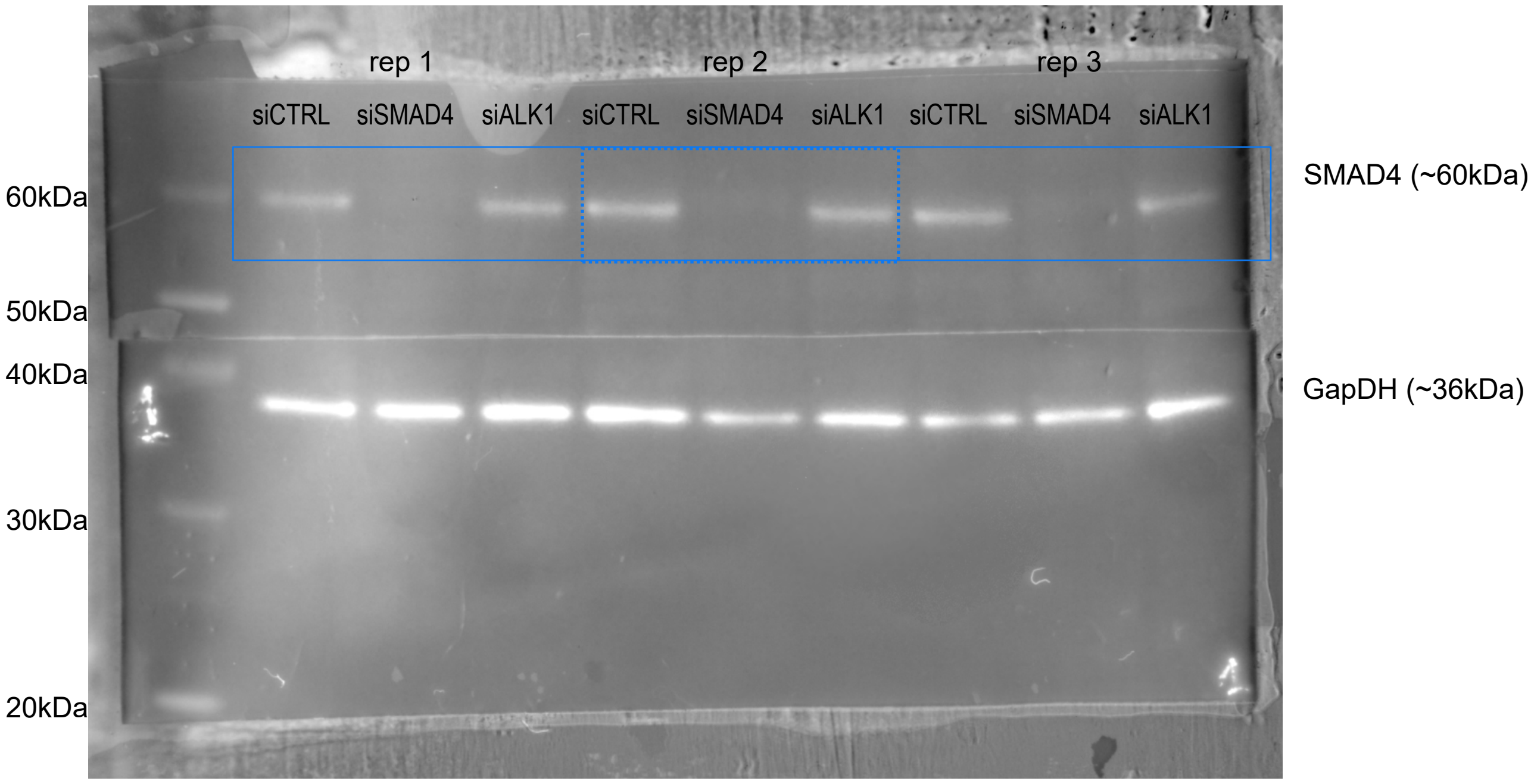

Supplement: Supplementary file 1 [file atv-46-e323908-s009.pdf]
