# Supplementary material for: Gene-Specific Endothelial Programs Drive AVM Pathogenesis in SMAD4 and ALK1 Loss-of-Function
Source: Arterioscler Thromb Vasc Biol. 2026 Jul 16;46(9):e323908. doi: 10.1161/ATVBAHA.125.323908 (PMC13506206; doi:10.1161/ATVBAHA.125.323908)
Supplement: Supplementary file 2 [file atv-46-e323908-s016.pdf]

# SUPPLEMENTAL MATERIALS

## Gene-specific endothelial programs drive AVM pathogenesis in *SMAD4* and *ALK1* loss-of-function

Authors: Dr. Olya Oppenheim[1,2,3], Dr. Wolfgang Giese[1,2], Dr. Hyojin Park[6], Dr. Elisabeth Baumann[1,2,3], Dr. Andranik Ivanov[3,4], Prof. Dieter Beule[3,4], Prof. Anne Eichmann[5,6,7], Prof. Holger Gerhardt[1,2,3,4]

Affiliations:

[1] Max Delbrück Center for Molecular Medicine in the Helmholtz Association, Berlin, Germany

[2] DZHK (German Center for Cardiovascular Research), Partnersite Berlin, Germany

[3] Charité Universitätsmedizin Berlin, Germany

[4] Berlin Institute of Health, Berlin, Germany

[5] Cardiovascular Research Center, Department of Internal Medicine, Yale University School of Medicine, New Haven, CT, USA

[6] Department of Molecular and Cellular Physiology, Yale University School of Medicine, New Haven, CT, USA

[7] PARCC, INSERM, Université de Paris, Paris, France

**Figure S1.1**

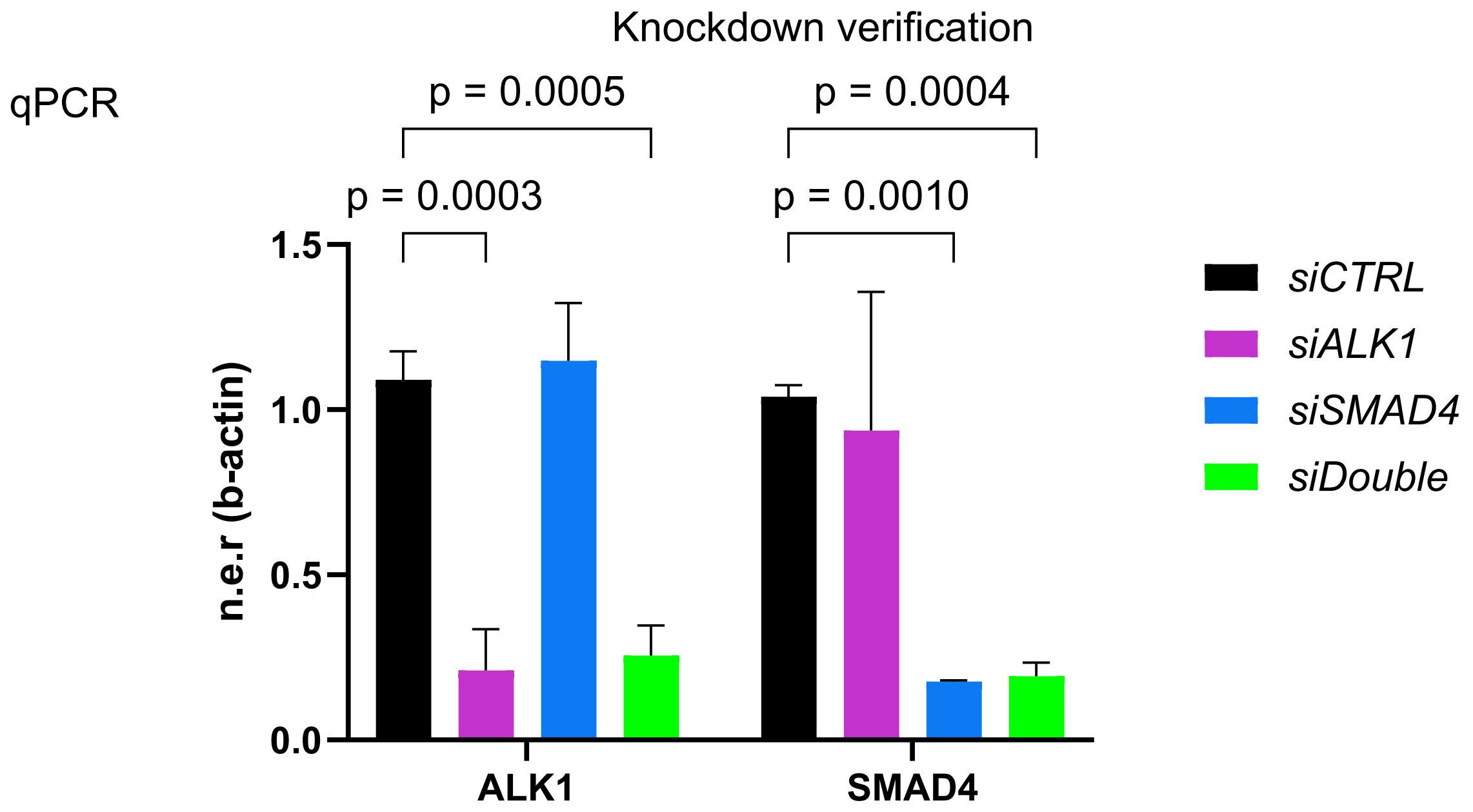

Knockdown verification
qPCR
p = 0.0005
p = 0.0004
p = 0.0003
p = 0.0010
1.5
1.0
0.5
0.0
n.e.r (b-actin)
ALK1
SMAD4
siCTRL
siALK1
siSMAD4
siDouble


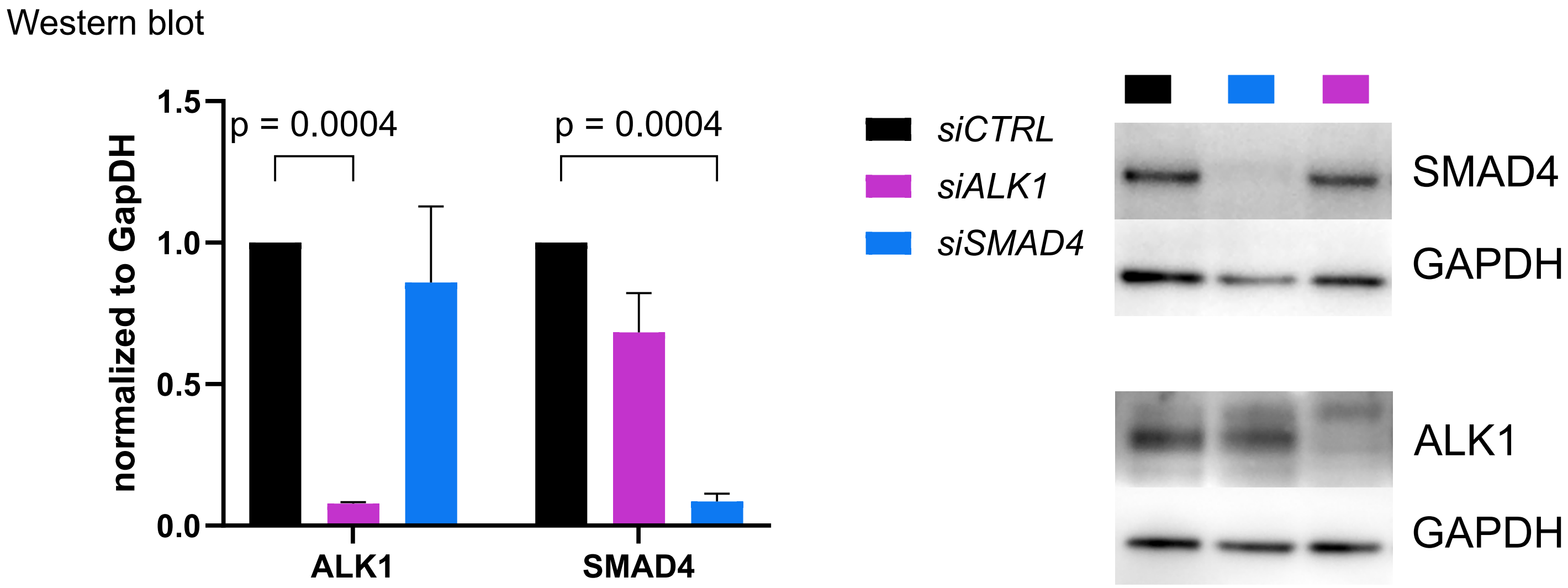

Western blot
1.5
1.0
0.5
0.0
normalized to GapDH
p = 0.0004
p = 0.0004
ALK1
SMAD4
siCTRL
siALK1
siSMAD4
SMAD4
GAPDH
ALK1
GAPDH

Figure S1.1:

Knockdown verification of *SMAD4*, *ALK1*. Top – qPCR analysis of *siCTRL* (black), *siSMAD4* (blue), *siALK1* (magenta), siDouble (lime), 48h post siRNA treatment. Log2FC values represent relative expression to b-actin. n=3 independent experiments. Bottom – Western blot analysis of *siCTRL*, *siSMAD4* and *siALK1* with representative images of blots, 48h post siRNA treatment. Bands normalized to GAPDH; Data presented as Mean ±SEM. n=3 independent experiments. Statistical analysis done using 2-way ANOVA with Dunnett's multiple comparisons test. Quantifcation data available in 01_statistics.xlsx. Raw blot images are included in WB.pdf

Figure S1.2

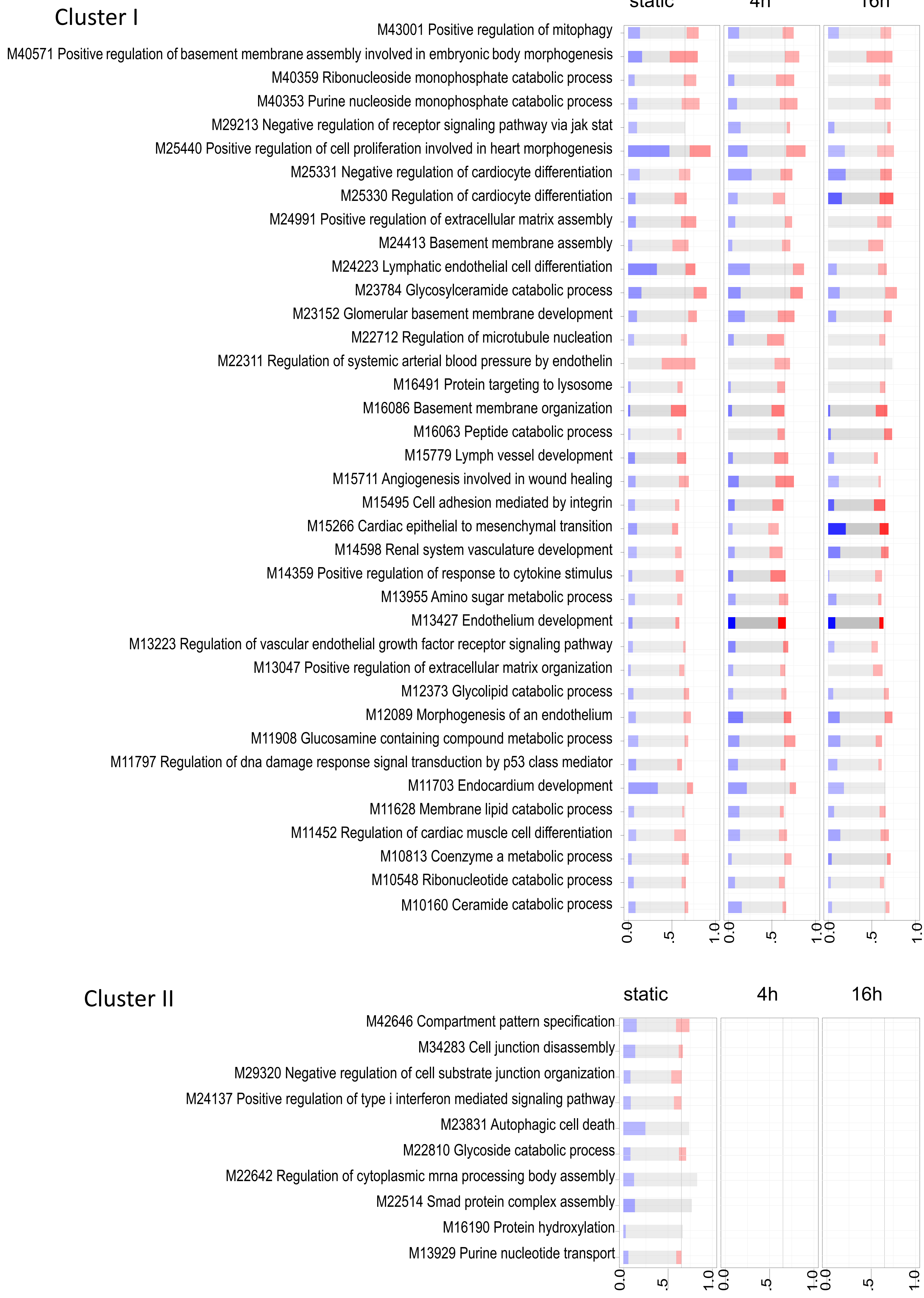
Cluster I
static
4h
16h
M43001 Positive regulation of mitophagy
M40571 Positive regulation of basement membrane assembly involved in embryonic body morphogenesis
M40359 Ribonucleoside monophosphate catabolic process
M40353 Purine nucleoside monophosphate catabolic process
M29213 Negative regulation of receptor signaling pathway via jak stat
M25440 Positive regulation of cell proliferation involved in heart morphogenesis
M25331 Negative regulation of cardiocyte differentiation
M25330 Regulation of cardiocyte differentiation
M24991 Positive regulation of extracellular matrix assembly
M24413 Basement membrane assembly
M24223 Lymphatic endothelial cell differentiation
M23784 Glycosylceramide catabolic process
M23152 Glomerular basement membrane development
M22712 Regulation of microtubule nucleation
M22311 Regulation of systemic arterial blood pressure by endothelin
M16491 Protein targeting to lysosome
M16086 Basement membrane organization
M16063 Peptide catabolic process
M15779 Lymph vessel development
M15711 Angiogenesis involved in wound healing
M15495 Cell adhesion mediated by integrin
M15266 Cardiac epithelial to mesenchymal transition
M14598 Renal system vasculature development
M14359 Positive regulation of response to cytokine stimulus
M13955 Amino sugar metabolic process
M13427 Endothelium development
M13223 Regulation of vascular endothelial growth factor receptor signaling pathway
M13047 Positive regulation of extracellular matrix organization
M12373 Glycolipid catabolic process
M12089 Morphogenesis of an endothelium
M11908 Glucosamine containing compound metabolic process
M11797 Regulation of dna damage response signal transduction by p53 class mediator
M11703 Endocardium development
M11628 Membrane lipid catabolic process
M11452 Regulation of cardiac muscle cell differentiation
M10813 Coenzyme a metabolic process
M10548 Ribonucleotide catabolic process
M10160 Ceramide catabolic process
0.0
.5
1.0
Cluster II
static
4h
16h
M42646 Compartment pattern specification
M34283 Cell junction disassembly
M29320 Negative regulation of cell substrate junction organization
M24137 Positive regulation of type i interferon mediated signaling pathway
M23831 Autophagic cell death
M22810 Glycoside catabolic process
M22642 Regulation of cytoplasmic mrna processing body assembly
M22514 Smad protein complex assembly
M16190 Protein hydroxylation
M13929 Purine nucleotide transport
0.0
.5
1.0

**Figure S1.2 - continued**

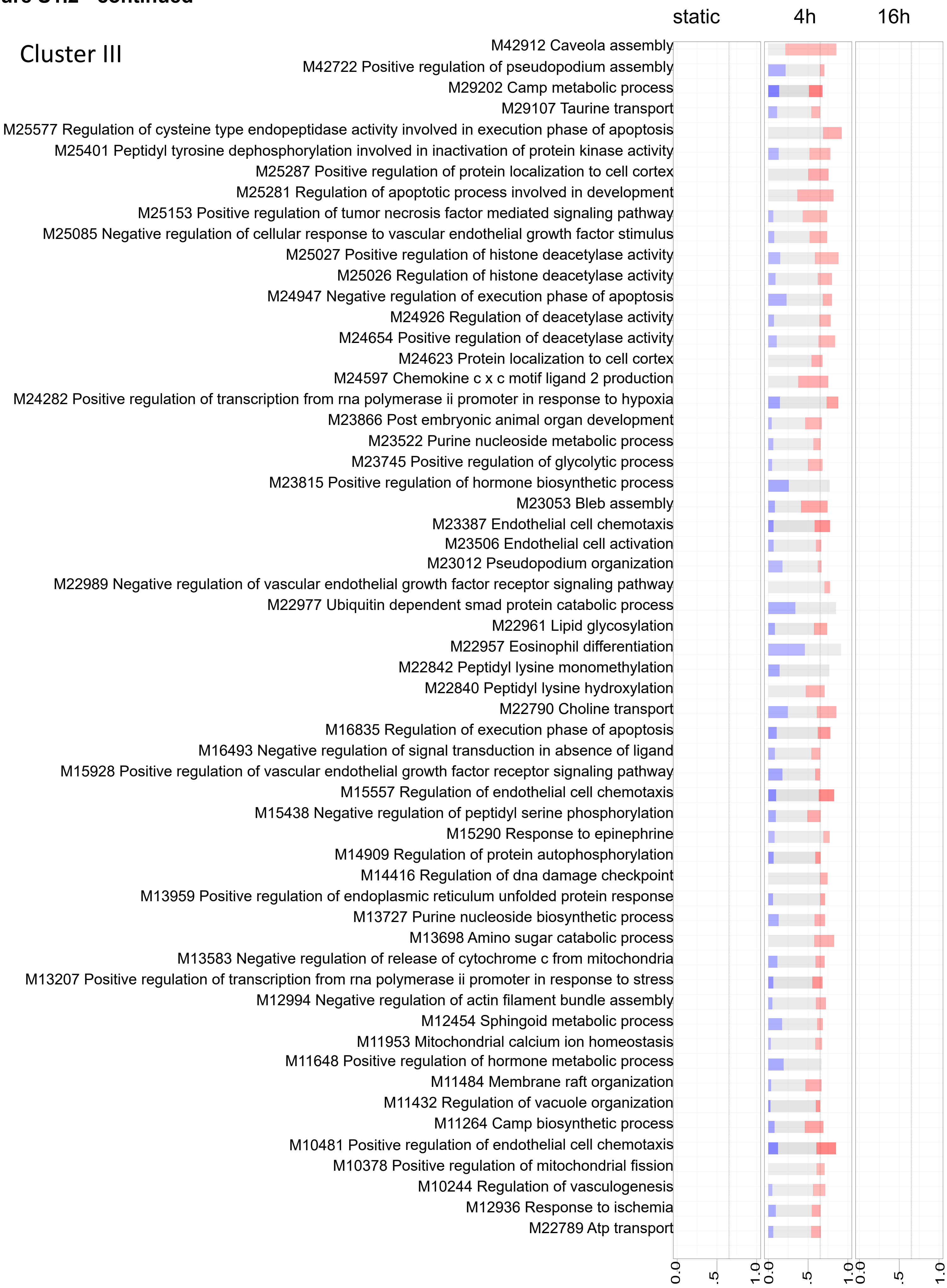

**Figure S1.2 - continued**

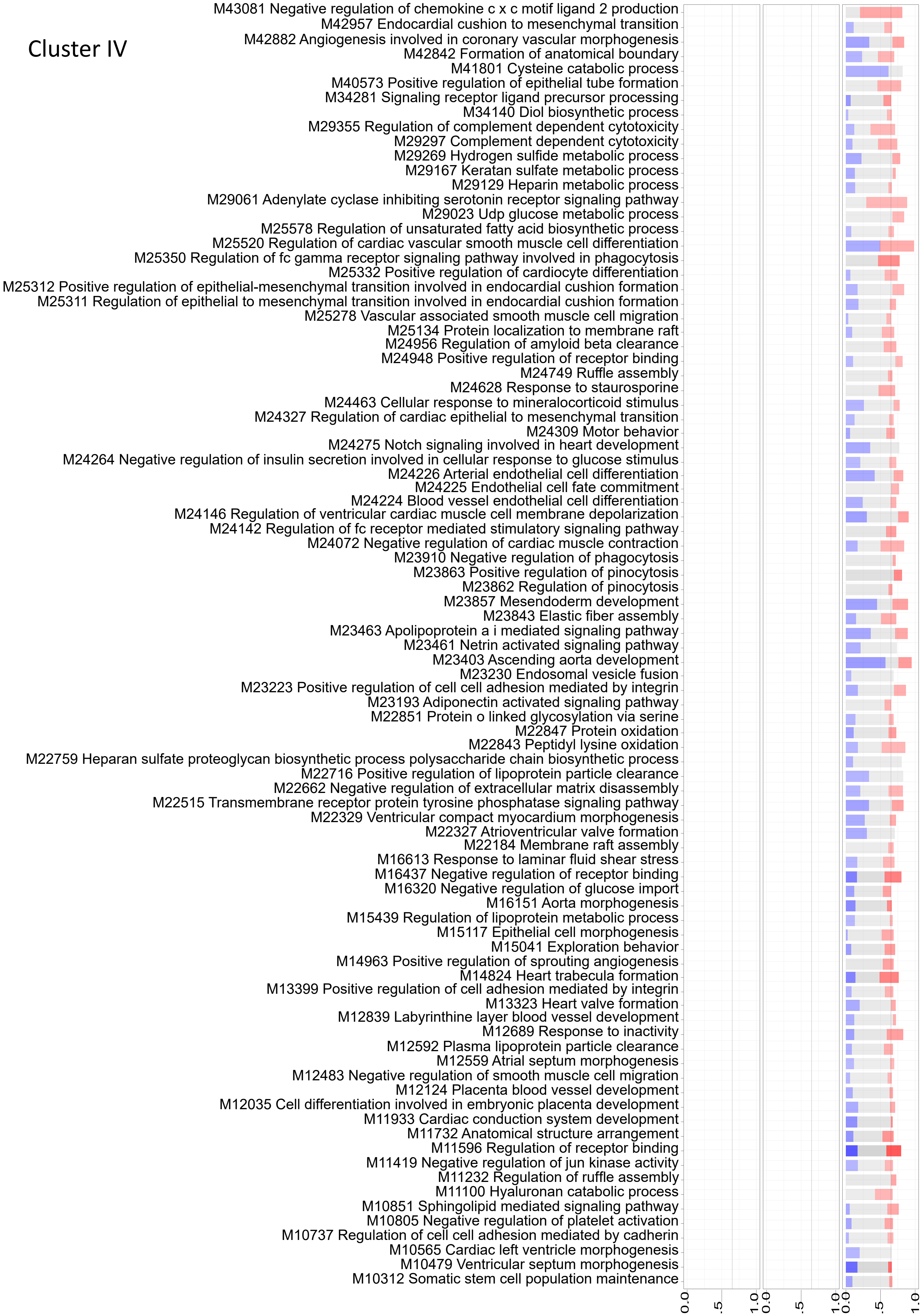

**Figure S1.2 - continued**

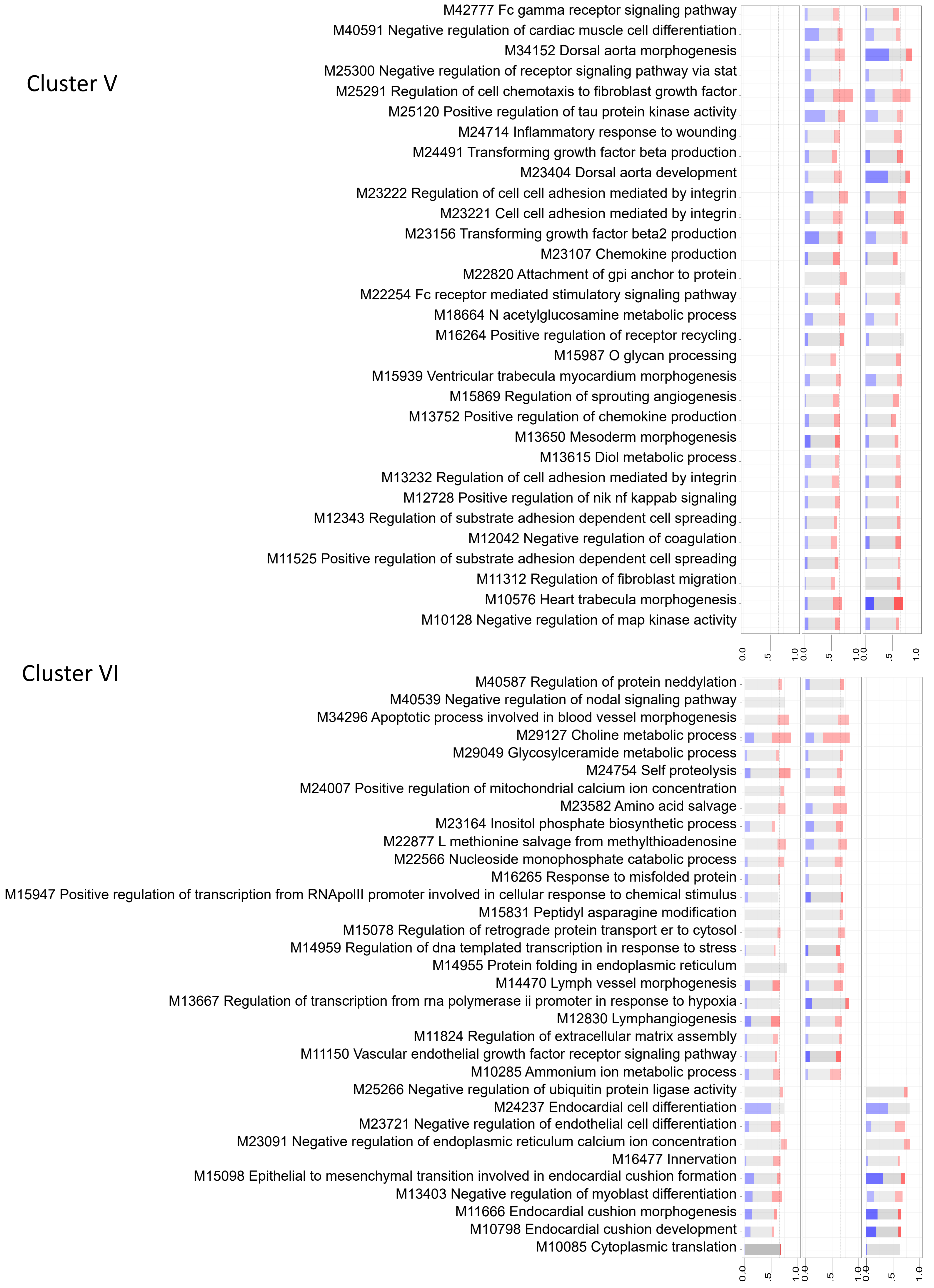

Figure S1.2

Full panel plot of GO_BP of *siSMAD4* vs. *siALK1* in static, 4h and 16h of LSS. GO_BPs are clustered according to their enrichment in the different flow conditions: Cluster I – enrichment in all flow conditions; Cluster II – enrichment in static only; Cluster III – enrichment in 4h LSS only; Cluster IV – enrichment in 16h LSS only; Cluster V – enrichment in both 4h and 16h LSS; Cluster VI – enrichment in static and 4h LSS **OR** 16h LSS.

**Figure S1.3**

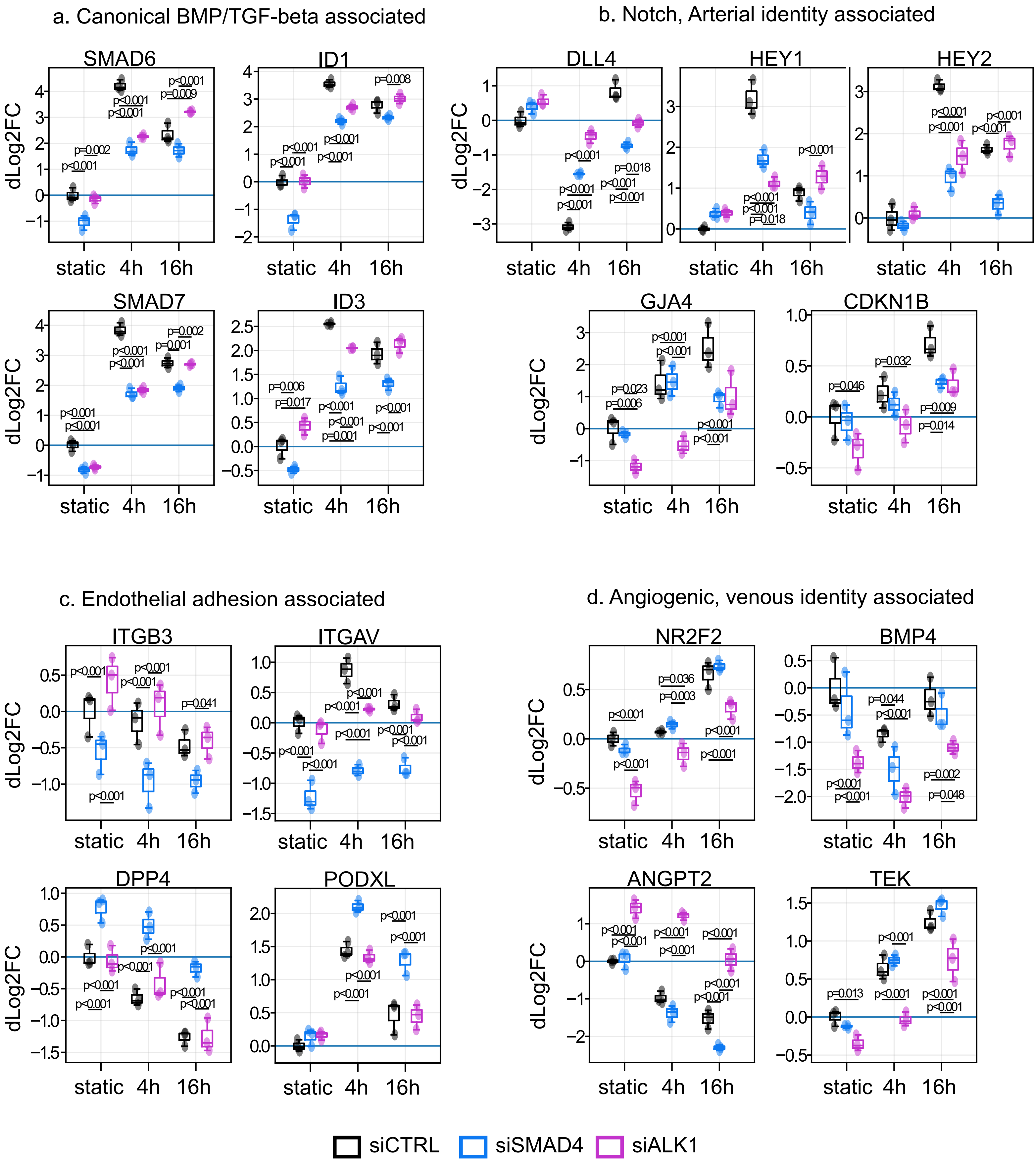

a. Canonical BMP/TGF-beta associated
b. Notch, Arterial identity associated
SMAD6
ID1
DLL4
HEY1
HEY2
SMAD7
ID3
GJA4
CDKN1B
c. Endothelial adhesion associated
d. Angiogenic, venous identity associated
ITGB3
ITGAV
NR2F2
BMP4
DPP4
PODXL
ANGPT2
TEK
dLog2FC
static
4h
16h
siCTRL
siSMAD4
siALK1

Figure S1.3

Expression patterns of individual genes over time (RNA-seq data). Y axis is Log2FC scale normalized to *siCTRL* static, X axis are flow conditions. Expression patterns of (a) Canonical BMP/TGF-beta associated genes; (b) Notch and Arterial identity associated genes; (c) Endothelial adhesion associated genes; and (d) Angiogenic and venous identity associated genes. *siCTRL* – black; *siSMAD4* – blue; *siALK1* – magenta. Adjusted p-values under 0.05 are indicated.

## Figure S2

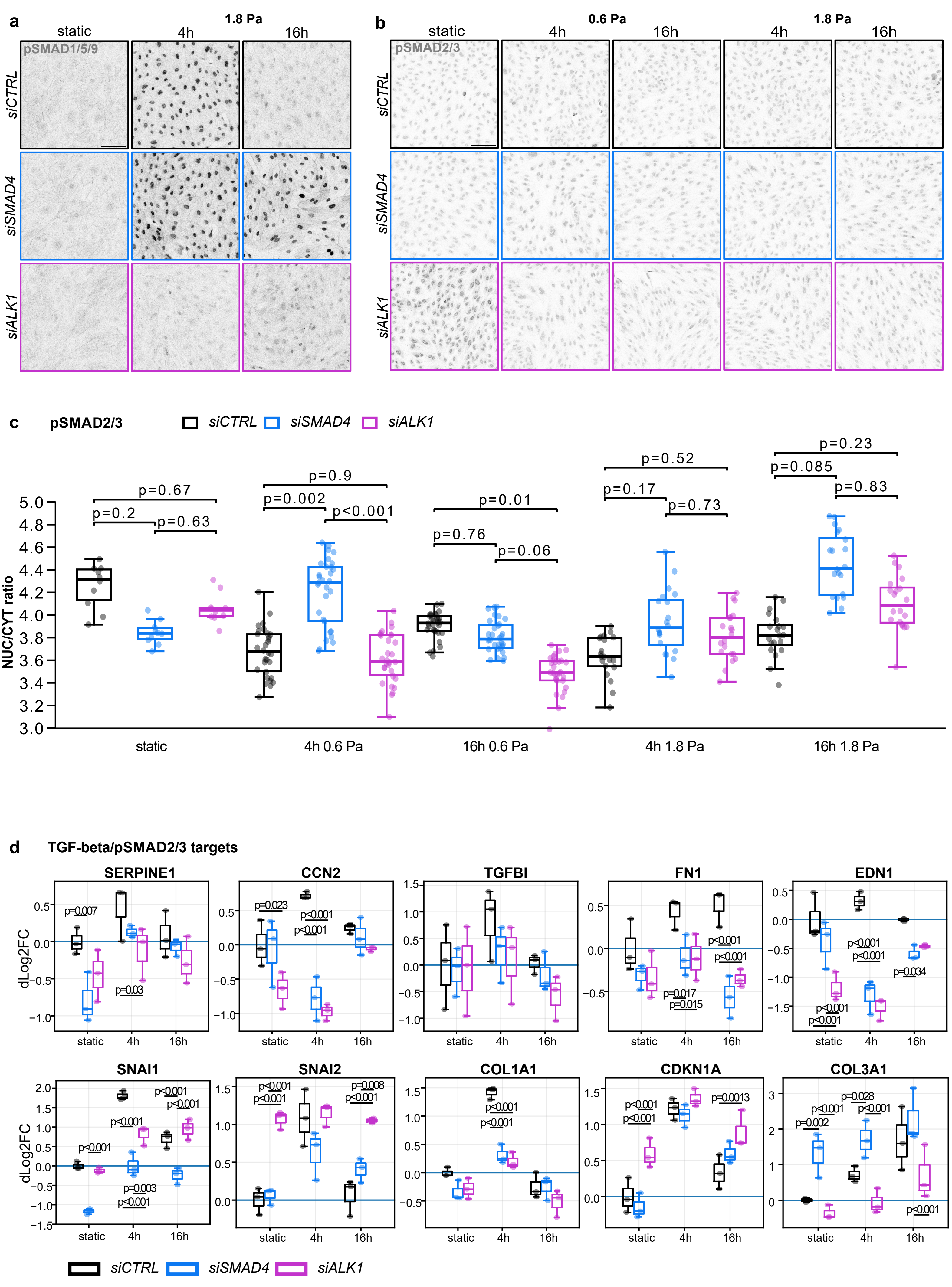

a
1.8 Pa
static
4h
16h
pSMAD1/5/9
siCTRL
siSMAD4
siALK1
b
0.6 Pa
1.8 Pa
static
4h
16h
pSMAD2/3
c
pSMAD2/3
siCTRL siSMAD4 siALK1
NUC/CYT ratio
p=0.67
p=0.2
p=0.63
p=0.9
p=0.002
p<0.001
p=0.01
p=0.76
p=0.06
p=0.52
p=0.17
p=0.73
p=0.23
p=0.085
p=0.83
static
4h 0.6 Pa
16h 0.6 Pa
4h 1.8 Pa
16h 1.8 Pa
d
TGF-beta/pSMAD2/3 targets
SERPINE1
CCN2
TGFBI
FN1
EDN1
SNAI1
SNAI2
COL1A1
CDKN1A
COL3A1
dLog2FC
static
4h
16h
siCTRL siSMAD4 siALK1

Figure S2

a. Representative pSMAD1/5/9 immunofluorescence images of siRNA-treated HUVECs after exposure to HSS (1.8 Pa). Scale bar 100µm.
b. Representative pSMAD2/3 immunofluorescence images of HUVECs after exposure to static, 4h and 16h, 0.6 and 1.8 Pa conditions. Scale bar 100µm.
c. Quantification of nuclear pSMAD2/3 signal at all conditions, normalized to cytosolic signal. Box- and scatter plots display the distribution of raw image-level measurements (≤10 images per experiment per condition). Statistical analysis was done on experimental means using 2-way ANOVA with Tukey's multiple comparison correction. Sample size: n_static=1; n_0.6_Pa=3 (both time points); n_1.8_Pa=2 (both time points). Full statistical analysis outputs are available in 02_statistics.xlsx
d. Individual expression patterns of TGF-beta/pSMAD2/3 targets under 0.6 Pa (LSS) flow (RNA-seq data). Y axis is Log2FC scale normalized to *siCTRL* static. Expression patterns of a. *siCTRL* – black; *siSMAD4* – blue; *siALK1* – magenta. Adjusted p-values under 0.05 are indicated.

## Figure S3

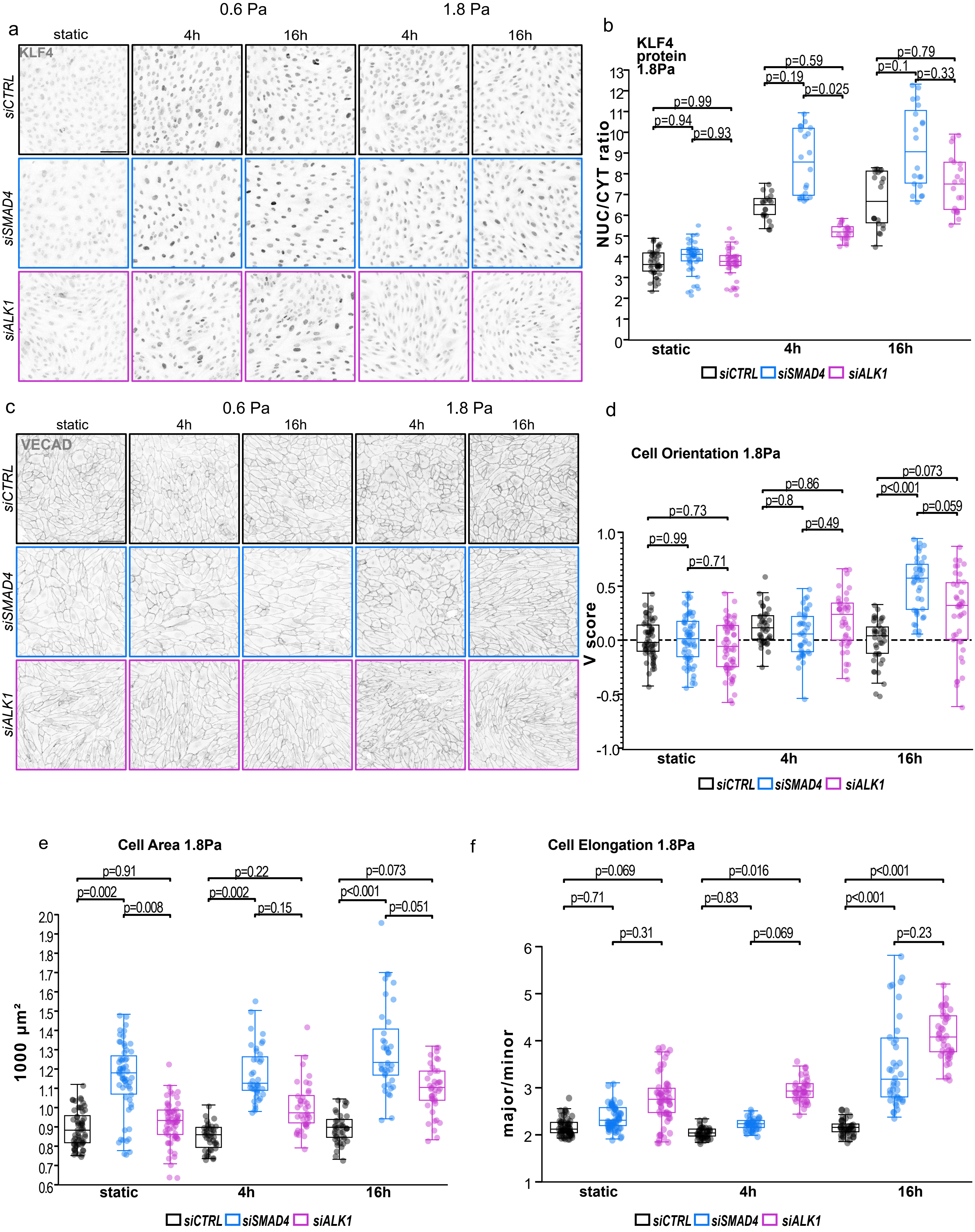

a
0.6 Pa
1.8 Pa
static
4h
16h
4h
16h
KLF4
siCTRL
siSMAD4
siALK1
b
KLF4
protein
1.8Pa
NUC/CYT ratio
p=0.99
p=0.94
p=0.93
p=0.59
p=0.19
p=0.025
p=0.79
p=0.1
p=0.33
static
4h
16h
siCTRL siSMAD4 siALK1
c
0.6 Pa
1.8 Pa
static
4h
16h
4h
16h
VECAD
siCTRL
siSMAD4
siALK1
d
Cell Orientation 1.8Pa
V score
p=0.73
p=0.99
p=0.71
p=0.86
p=0.8
p=0.49
p=0.073
p<0.001
p=0.059
static
4h
16h
siCTRL siSMAD4 siALK1
e
Cell Area 1.8Pa
1000 µm²
p=0.91
p=0.002
p=0.008
p=0.22
p=0.002
p=0.15
p=0.073
p<0.001
p=0.051
static
4h
16h
siCTRL siSMAD4 siALK1
f
Cell Elongation 1.8Pa
major/minor
p=0.069
p=0.71
p=0.31
p=0.016
p=0.83
p=0.069
p<0.001
p<0.001
p=0.23
static
4h
16h
siCTRL siSMAD4 siALK1

Figure S3

a. Representative *KLF4* immunofluorescence images of HUVECs after exposure to static, 4h and 16h, 0.6 and 1.8 Pa conditions. Scale bar 100µm.

b. Quantification of nuclear *KLF4* signal at 1.8 Pa, normalized to cytosolic signal. Box- and scatter plots display the distribution of raw image-level measurements (≤10 images per experiment per condition). n=2 independent experiments. Statistical analysis was done on experimental means using 2-way ANOVA with Tukey's multiple comparison correction.

c. Representative VEcad immunofluorescence images of HUVECs after exposure to static, 4h and 16h, 0.6 and 1.8 Pa conditions. Scale bar 100µm.

d. – f. Statistical analysis of cell orientation (d), cell area (e) and cell elongation (f) of *siCTRL-*, *siSMAD4*- and *siALK1*-treated HUVECs at static, 4h and 16h at 1.8Pa (HSS). ). Box- and scatter plots display the distribution of raw image-level measurements (≤10 images per experiment per condition). Statistical analysis was done on experimental means using 2-way ANOVA with Tukey's multiple comparison correction. Full statistical analysis outputs are available in 03_statistics.xlsx

## Figure S4

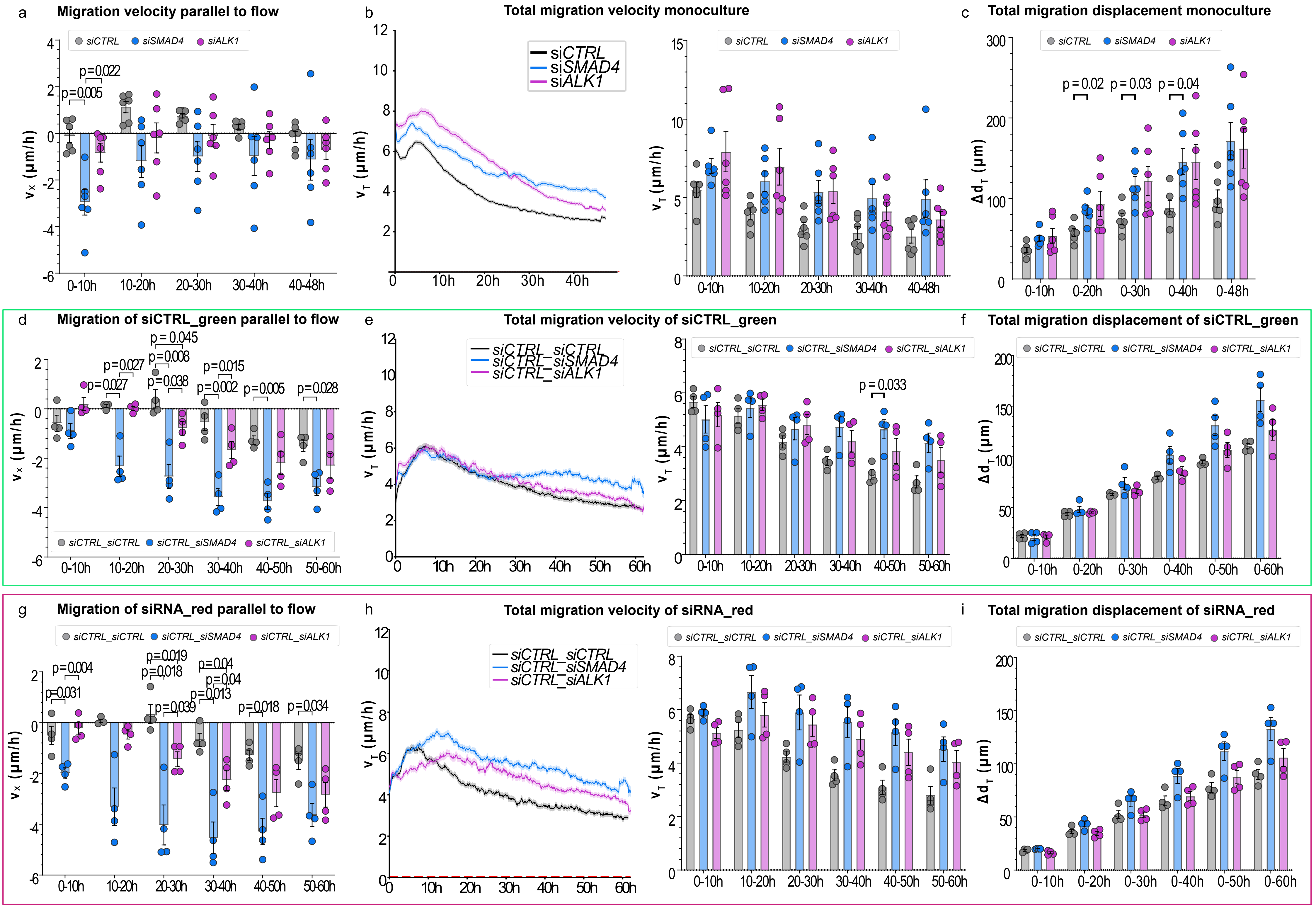

a
Migration velocity parallel to flow
siCTRL siSMAD4 siALK1
p=0.022
p=0.005
$v_x$ (µm/h)
0-10h 10-20h 20-30h 30-40h 40-48h
b
Total migration velocity monoculture
siCTRL
siSMAD4
siALK1
$v_T$ (µm/h)
0 10h 20h 30h 40h
0-10h 10-20h 20-30h 30-40h 40-48h
c
Total migration displacement monoculture
p = 0.02 p = 0.03 p = 0.04
$\Delta d_T$ (µm)
0-10h 0-20h 0-30h 0-40h 0-48h
d
Migration of siCTRL_green parallel to flow
p=0.045
p=0.027
p=0.008
p=0.015
p=0.027
p=0.038
p=0.002
p=0.005
p=0.028
siCTRL_siCTRL siCTRL_siSMAD4 siCTRL_siALK1
0-10h 10-20h 20-30h 30-40h 40-50h 50-60h
e
Total migration velocity of siCTRL_green
p = 0.033
0 10h 20h 30h 40h 50h 60h
f
Total migration displacement of siCTRL_green
0-10h 0-20h 0-30h 0-40h 0-50h 0-60h
g
Migration of siRNA_red parallel to flow
p=0.004
p=0.031
p=0.019
p=0.018
p=0.039
p=0.04
p=0.04
p=0.013
p=0.018
p=0.034
h
Total migration velocity of siRNA_red
i
Total migration displacement of siRNA_red

Figure S4

a-c. Migration analysis of cells in monoculture. (a) Quantification of mean migration velocity parallel to flow direction of *siCTRL-*, *siSMAD4-* or *siALK1*-treated HUVECs. (b) Total migration velocity plot and Quantification of *siCTRL-*, *siSMAD4-* or *siALK1*-treated HUVECs. (c) Total migration displacement analysis of *siCTRL-*, *siSMAD4-* or *siALK1*-treated HUVECs.

d-f. Migration analysis of *siCTRL*_green cells in mosaic co-cultures. (d) Quantification of mean migration velocity parallel to flow direction of *siCTRL*-treated HUVECs when co-cultured either with themselves *(siCTRL_siCTRL*, gray), or with *siSMAD4-* or *siALK1*-treated HUVECs (*siCTRL_siSMAD4*, blue and *siCTRL_siALK1*, magenta). (e) Total migration velocity plot and Quantification of *siCTRL*-treated HUVECs when co-cultured either with themselves *(siCTRL_siCTRL*, gray), or with *siSMAD4-* or *siALK1*-treated HUVECs (*siCTRL_siSMAD4*, blue and *siCTRL_siALK1*, magenta). (f) Total migration displacement analysis of *siCTRL*-treated HUVECs when co-cultured either with themselves *(siCTRL_siCTRL*, gray), or with *siSMAD4-* or *siALK1-* treated HUVECs (*siCTRL_siSMAD4*, blue and *siCTRL_siALK1*, magenta).

g-i. Migration analysis of *siRNA*_red cells in mosaic co-cultures. (g) Quantification of mean migration velocity parallel to flow direction of red fraction of *siRNA*-treated HUVECs when co-cultured with *siCTRL*_green *(siCTRL_siCTRL*, gray; *siCTRL_siSMAD4*, blue; or *siCTRL_siALK1*, magenta). (h) Total migration velocity plot and Quantification of red fraction of *siRNA*-treated HUVECs when co-cultured with *siCTRL*_green *(siCTRL_siCTRL*, gray; *siCTRL_siSMAD4*, blue; or *siCTRL_siALK1*, magenta). (i) Total migration displacement analysis of *siRNA*-treated HUVECs when co-cultured with *siCTRL*_green *(siCTRL_siCTRL*, gray; *siCTRL_siSMAD4*, blue; or *siCTRL_siALK1*, magenta).

Statistical analysis was done using 2-way ANOVA with Holm-Šídák multiple comparison correction. Full statistical outputs available in 04_statistics.xlsx.

**Figure S5**

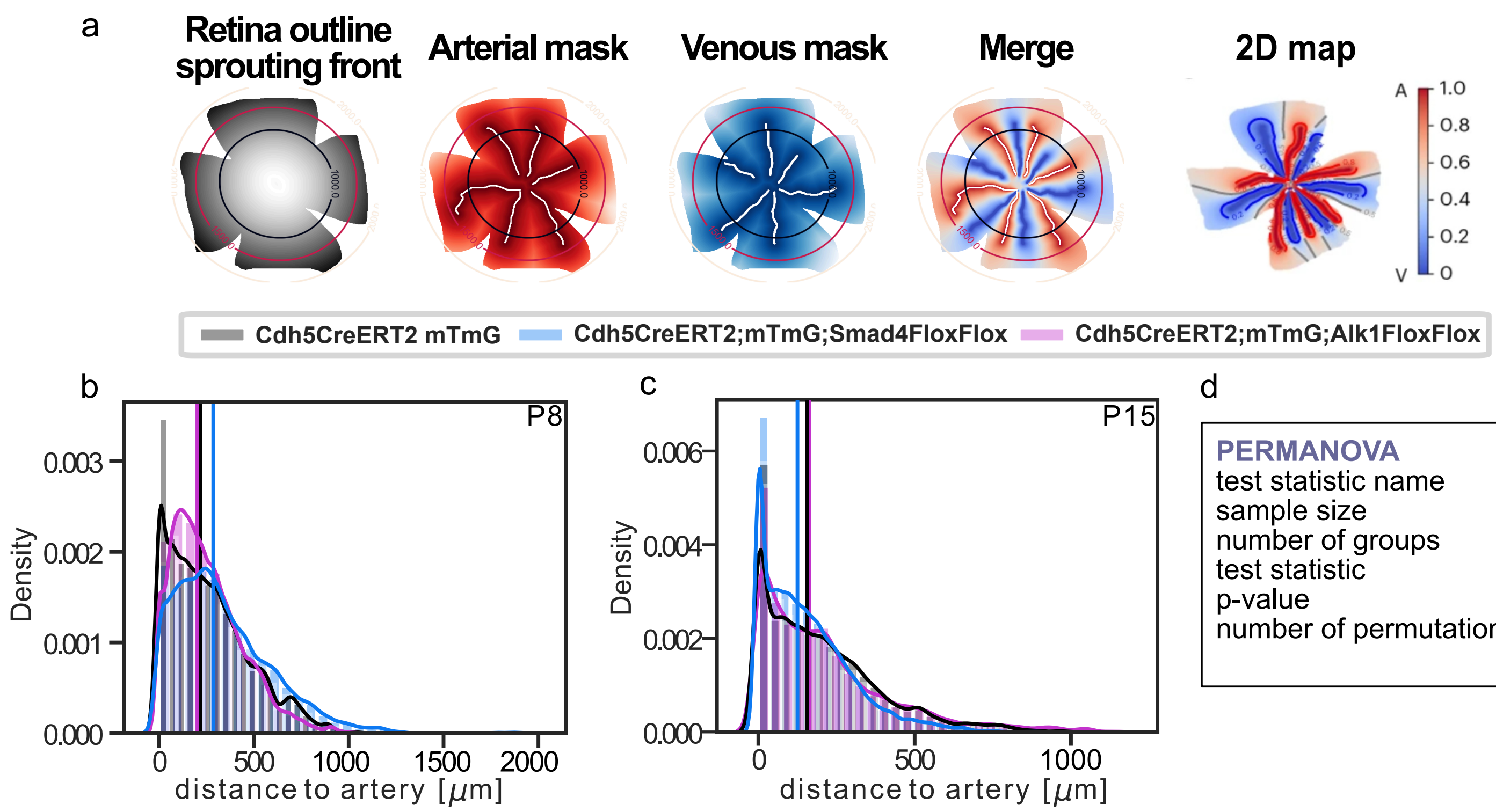


d

| PERMANOVA | |
|---|---|
| test statistic name | pseudo-F |
| sample size | 54 |
| number of groups | 6 |
| test statistic | 11.82169 |
| p-value | 0.001 |
| number of permutations | 999 |

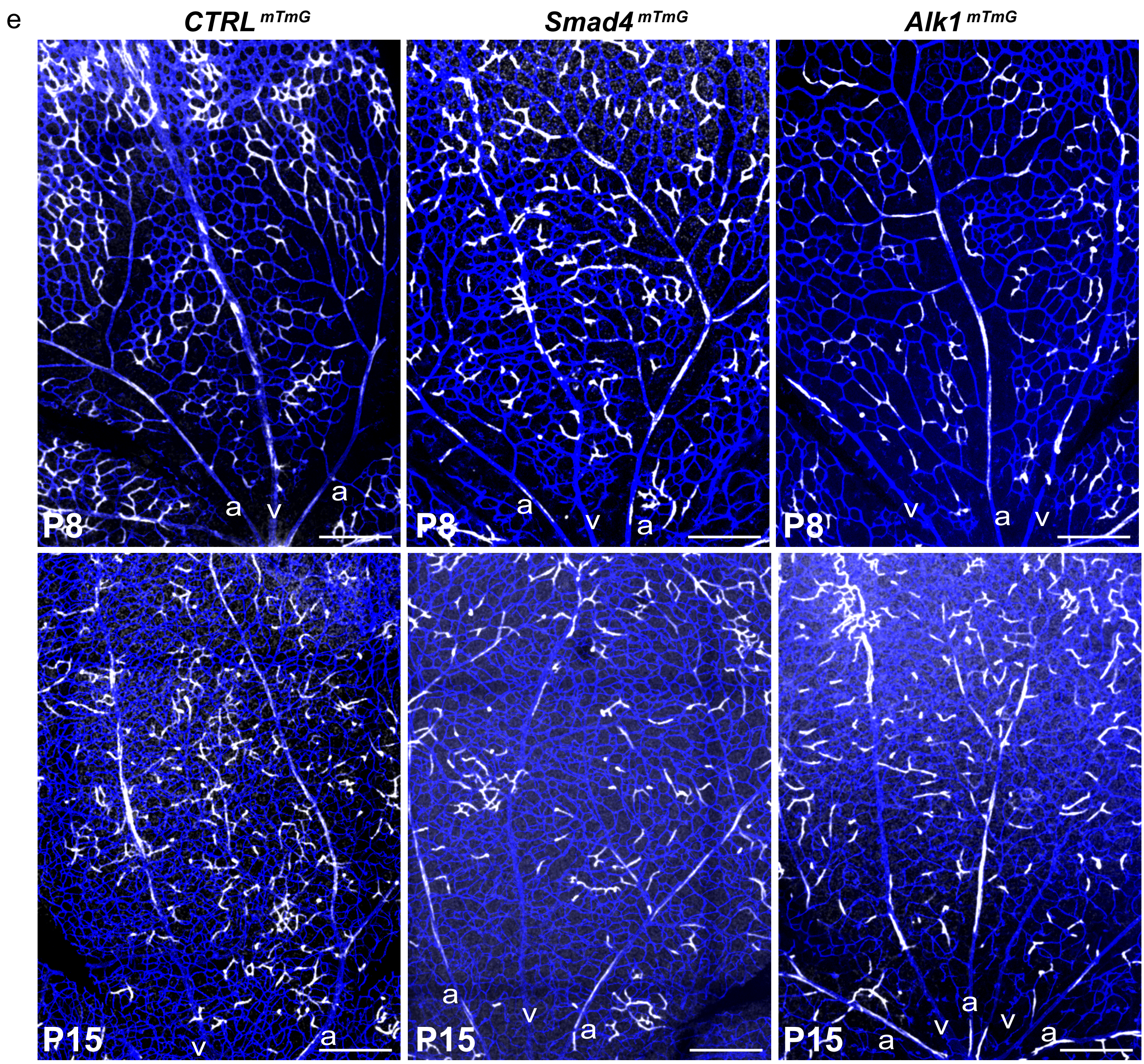

**Figure S5**

a. Analysis overview: two-dimensional masks of retina outline, arteries and veins in flat-mounted retinae, result in a 2D map. Each $GFP^+$ EC's coordinates are based on its location relative to the vein (0.0) and artery (1.0) and the radial distance from the optic nerve to the sprouting front. Kernel Density Estimate (KDE) plots represent the cumulative location of all $GFP^+$ EC across all retinae for each genotype.

b. – c. Density histogram of $GFP^+$ pixel distribution relative to the arteries at P8 (b) and P15 (c), respectively. Vertical lines represent means of each genotype (gray – $CTRL^{mTmG}$; blue – $Smad4^{mTmG}$; magenta – $Alk1^{mTmG}$).

d. PERMANOVA analysis output of PC1 and PC2.

e. Higher magnification images of retina sections shown in Figure 5. Scale bar 250µm. a-artery; v-vein.

Figure S6

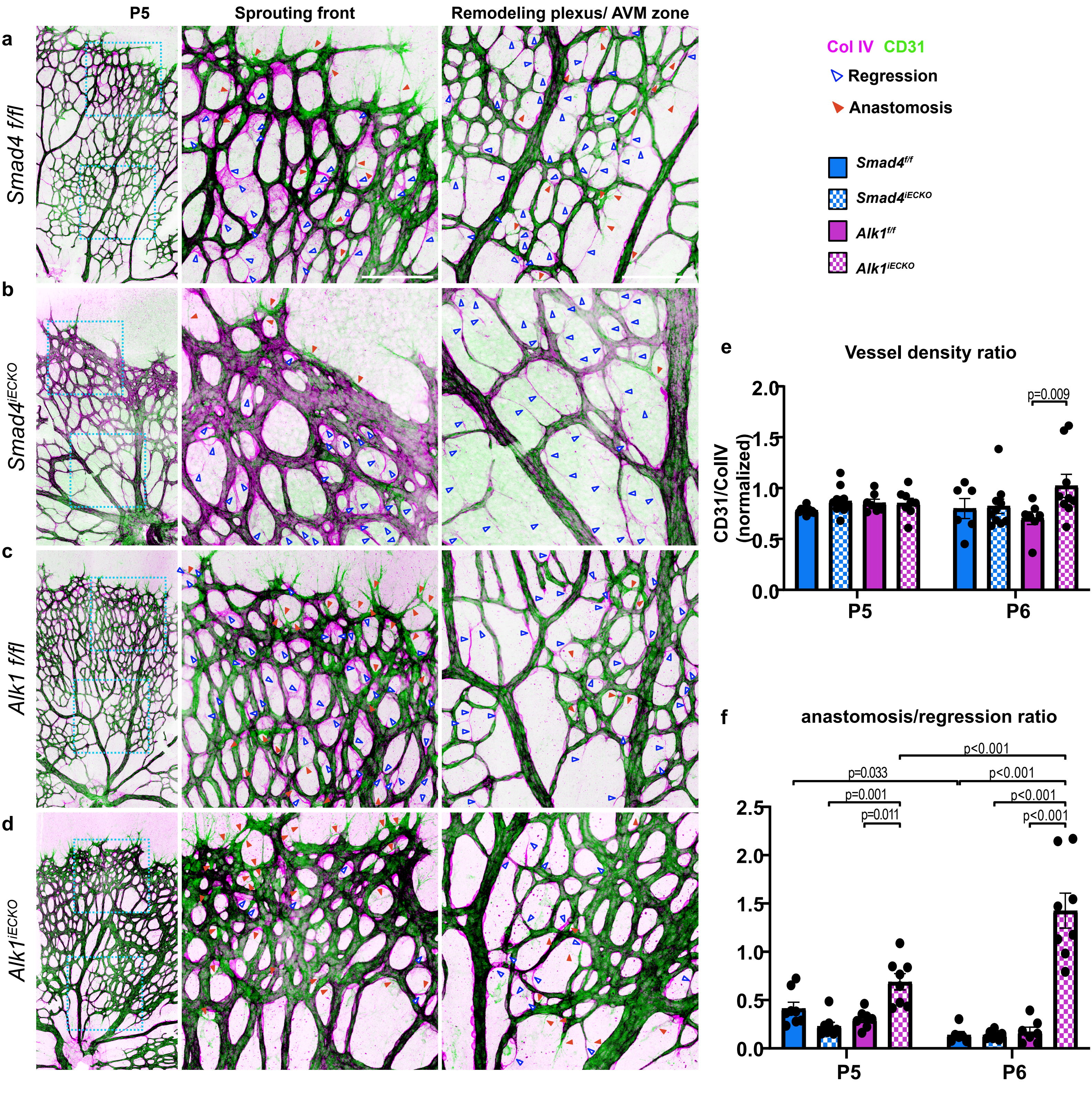

P5
Sprouting front
Remodeling plexus/ AVM zone
a
Smad4 f/fl
b
Smad4iECKO
c
Alk1 f/fl
d
Alk1iECKO
Col IV CD31
Regression
Anastomosis
Smad4f/f
Smad4iECKO
Alk1f/f
Alk1iECKO
e
Vessel density ratio
CD31/ColIV (normalized)
2.0
1.5
1.0
0.5
0.0
p=0.009
P5
P6
f
anastomosis/regression ratio
2.5
2.0
1.5
1.0
0.5
0.0
p<0.001
p=0.033
p<0.001
p=0.001
p<0.001
p=0.011
p<0.001
P5
P6

Figure S6

a–d. Microscopy images of P5 retinae of *Smad4* $^{fl/fl}$ (a), *Smad4*$^{iECKO}$ (b), *Alk1* $^{fl/fl}$ (c), *Alk1*$^{iECKO}$ (d). Light blue dashed rectangles mark areas of sprouting front (SF) and remodeling plexus/AVM zone (RP) on the right of each image. Scale bar 100µm. Anastomosis events marked in orange triangles; Regression events marked in dark blue unfilled triangles.

e–f. Vessel density (vessel volume/ROI volume x100%) (e); and anastomosis/regression events ratios (f) in littermate controls (full-color bars) and KO retinae (dotted bars). Collection time points (P5 or P6) are annotated under each group. 2-way ANOVA test was used to determine statistical significance with Tukey's multiple correction. Data are represented as mean ± SEM. Full statistical outputs available in 06_statistics.xlsx.

Supplementary movies:

Live imaging of endothelial migration under flow.

**S1-3.** Representative time lapse imaging of HUVECs labeled with SPY505 and exposed to LSS for 48h. 1. *siCTRL*; 2. *siSMAD4*; 3. *siALK1*. Frame interval – 7.5 minutes.

**S4-6.** Representative time lapse imaging of HUVECs labeled with CellTracker Green (*siCTRL*) and CellTracker Red (*siCTRL/siSMAD4/siALK1*) and exposed to LSS for 62h. 4. *siCTRL* mosaic (green + red); 5. *siCTRL* green+*siSMAD4* red; 6. *siCTRL* green+*siALK1* red.

# Major Resources Table

## Mouse Lines

| Strain | Vendor or Source | Background Strain | Sex | Diet, housing&husbandry conditions | Persistent ID / URL |
|---|---|---|---|---|---|
| *$Alk1^{f/f}$* | Dr. S.Paul Oh, Barrow Neurological Institute, Phoenix, Arizona | 129/C57BL/6J | M &F | Mice were fed a standard laboratory diet using Inotiv Teklad 2018S Sterilizable Rodent diet. Mice were housed in the Yale Animal Resources Center (YARC) facilities under a standard 12-hour light/12-hour dark cycle (typically lights on at 7:00 AM and off at 7:00 PM, or reversed depending on the room) to support circadian rhythms. Housing conditions were maintained at 65–75°F with 40–60% humidity. | https://pubmed.ncbi.nlm.nih.gov/19805914/ |
| *$Smad4^{f/f}$* | JAX (Strain# 017462) | C57BL/6J | M &F | | https://www.jax.org/strain/017462 |
| *mTmG* | JAX (Strain# 007676) | C57BL/6J | M &F | | https://www.jax.org/strain/007676# |
| *Cdh5 $Cre^{ERT2}$* | Dr. Ralf Adams, Max Planck Institute for Molecular Biomedicine, Münster, Germany | C57BL/6J | M& F | | ttps://pubmed.ncbi.nlm.nih.gov/20445537/ Taconic Biosciences, Model #: 13073 |

## Mouse Models Used in This Study

| **Strain** | **Background Strain** | **Sex** | **Breeding scheme** |
|---|---|---|---|
| *$Alk1^{f/f}$ Cdh5 $Cre^{ERT2}$ mTmG* | C57BL/6J | M&F | Generated with *$Alk1^{f/f}$, Cdh5 $Cre^{ERT2}$, mTmG*<br>F1 : *Cdh5 $Cre^{ERT2}$ X mTmG*<br>F2 : *$Alk1^{f/f}$ X Cdh5 $Cre^{ERT2}$ mTmG*<br>F3 *: $Alk1^{f/f}$ Cdh5 $Cre^{ERT2}$ mTmG* |
| *$Smad4^{f/f}$ Cdh5 $Cre^{ERT2}$ mTmG* | C57BL/6J | M&F | Generated with *$Smad4^{f/f}$, Cdh5 $Cre^{ERT2}$, mTmG*<br>F1 : *Cdh5 $Cre^{ERT2}$ X mTmG*<br>F2 : *$Smad4^{f/f}$ X Cdh5 $Cre^{ERT2}$ mTmG*<br>F3 : *$Smad4^{f/f}$ Cdh5 $Cre^{ERT2}$ mTmG* |
| *$Alk1^{f/f}$ Cdh5 $Cre^{ERT2}$* | C57BL/6J | M&F | Generated with *$Alk1^{f/f}$, Cdh5 $Cre^{ERT2}$*<br>F1 : *$Alk1^{f/f}$ X Cdh5 $Cre^{ERT2}$*<br>F2 *: $Alk1^{f/f}$ Cdh5 $Cre^{ERT2}$* |
| *$Smad4^{f/f}$ Cdh5 $Cre^{ERT2}$* | C57BL/6J | M&F | Generated with *$Smad4^{f/f}$, Cdh5 $Cre^{ERT2}$*<br>F1 : *$Smad4^{f/f}$ X Cdh5 $Cre^{ERT2}$*<br>F2 *: $Smad4^{f/f}$ Cdh5 $Cre^{ERT2}$* |
| *Cdh5 $Cre^{ERT2}$ mTmG* | C57BL/6J | M&F | Generated with *Cdh5 $Cre^{ERT2}$, mTmG*<br>F1 : *Cdh5 $Cre^{ERT2}$ X mTmG* |

DOI [to be added]

## Antibodies

| Target antigen | Vendor or Source | Catalog # | Working concentration | Persistent ID / URL |
|---|---|---|---|---|
| | | ***In vitro IF*** | | |
| VEcadherin | R&D Systems | AF938 | 1:1000 | https://www.rndsystems.com/products/human-ve-cadherin-antibody_af938 |
| GM130 | BD Bioscience | 610822 | 1:500 | https://www.bdbiosciences.com/en-de/products/reagents/microscopy-imaging-reagents/immunofluorescence-reagents/purified-mouse-anti-gm130.610822?tab=product_details |
| pSMAD159 | Cell Signaling | 13820S | 1:500 | https://www.cellsignal.com/products/primary-antibodies/phospho-smad1-ser463-465-smad5-ser463-465-smad9-ser465-467-d5b10-rabbit-monoclonal-antibody/13820?srsltid=AfmBOoo5FnI_TsfF7sJLPxdwfZukovTt954A_MldzwSKdSj-a84KTiy9 |
| pSMAD23 | Proteintech | 80427-2-RR | 1:500 | https://www.ptglab.com/products/Phospho-SMAD2-Ser465-467-Antibody-80427-2-RR.htm |
| KLF4 | Sigma Aldrich | HOA002926 | 1:500 | https://www.sigmaaldrich.com/DE/en/product/sigma/hpa002926?srsltid=AfmBOorL51iyJLpnhSoihzJTP1bfVGlzBwqt0PIL98Ik519kZfO_ORol |
| anti-goat IgG 568 | Thermo fisher | A11057 | 1:400 | https://www.thermofisher.com/antibody/product/Donkey-anti-Goat-IgG-H-L-Cross-Adsorbed-Secondary-Antibody-Polyclonal/A-11057 |
| anti-mouse IgG 488 | Thermo fisher | A21202 | 1:400 | https://www.thermofisher.com/antibody/product/Donkey-anti-Mouse-IgG-H-L-Highly-Cross-Adsorbed-Secondary-Antibody-Polyclonal/A-21202 |
| anti-rabbit IgG 647 | Thermo fisher | A31573 | 1:400 | https://www.thermofisher.com/antibody/product/Donkey-anti-Rabbit-IgG-H-L-Highly-Cross-Adsorbed-Secondary-Antibody-Polyclonal/A-31573 |
| | | ***In vivo IF*** | | |
| Isolectin B4 | Thermo fisher | 132450 | 1:400 | https://www.thermofisher.com/order/catalog/product/de/en/I32450 |
| GFP polyclonal antibody Alexa Fluor 488 | Thermo fisher | A-21311 | 1:1000 | https://www.thermofisher.com/antibody/product/GFP-Antibody-Polyclonal/A-21311 |
| CD31 | BD Bioscience | 553370 | 1:200 | https://www.bdbiosciences.com/en-dk/products/reagents/microscopy-imaging-reagents/immunohistochemistry-reagents/purified-rat-anti-mouse-cd31.553370?tab=product_details |
| Col IV | Bio-Rad | 2150-1470 | 1:400 | |
| Anti-rat Alexa Fluor 488 | Thermo fisher | A21208 | 1:500 | https://www.thermofisher.com/antibody/product/Donkey-anti-Rat-IgG-H-L-Highly-Cross-Adsorbed-Secondary-Antibody-Polyclonal/A-21208 |
| Anti-rabbit Alexa Fluor 568 | Donkey, Thermo fisher | A10042 | 1:500 | https://www.thermofisher.com/antibody/product/Donkey-anti-Rabbit-IgG-H-L-Highly-Cross-Adsorbed-Secondary-Antibody-Polyclonal/A10042 |

DOI [to be added]

| WB | | | | |
|---|---|---|---|---|
| SMAD 4 | Cell Signaling | 38454S | 1:1000 | https://www.cellsignal.com/products/primary-antibodies/smad4-d3m6u-rabbit-monoclonal-antibody/38454?site-search-type=Products&N=4294956287&Ntt=38454t&fromPage=plp&_requestid=186987 |
| ALK1 | Biosynth | 70R-49334 | 1:500 | https://www.biosynth.com/p/70R-49334/alk1-antibody |
| GAPDH | Cell Signaling | 971669 | 1:1000 | https://www.cellsignal.com/products/primary-antibodies/gapdh-d4c6r-mouse-mab/97166 |
| ECL anti-mouse IgG HRP | GE Heatlhcare | NA931V | 1:5000 | https://www.cytivalifesciences.com/en/us/shop/protein-analysis/blotting-and-detection/blotting-standards-and-reagents/amersham-ecl-hrp-conjugated-antibodies-p-06260 |
| anti-rabbit IgG HRP | Cell Signaling | 7074S | 1:5000 | https://www.cellsignal.de/products/secondary-antibodies/anti-rabbit-igg-hrp-linked-antibody/7074 |

### Cultured Cells

| Name | Vendor or Source | Sex (F, M, or unknown) | Persistent ID / URL |
|---|---|---|---|
| HUVECs | Promocell | Mixed donor (M+F) | Cat.No. C-12203 |

### Data & Code Availability

| Description | Source / Repository | Persistent ID / URL |
|---|---|---|
| Raw RNAseq reads | GEO database | GSE282952 |
| Python scripts | Github | https://github.com/gerhardt-lab/ |

### Analysis and statistics files

| Description | Persistent ID / URL |
|---|---|
| GO_BP: biological proccess for siSMAD4/vs_siALK1 in static, 4h and 16h if flow exposure Tha analysis was filtered out GO terms that had an AUC below 0.65, and padj values above 0.05 | Data Set 1.xlsx |
| DEG tables filtered through Log2FC of either above 0.5 or below -0.5, and padj of 0.05 | Data Set 2.xlsx |
| Statistical outputs for Fig. S1.1 | 01_statistics.xlsx |
| Statistical outputs for Fig. 2 | 02_statistics.xlsx |
| Statistical outputs for Fig. 3 | 03_statistics.xlsx |
| Statistical outputs for Fig. 4 | 04_statistics.xlsx |
| Statistical outputs for Fig. 5 | 05_statistics.xlsx |
| Statistical outputs for Fig. 6 | 06_statistics.xlsx |

DOI [to be added]

**ARRIVE GUIDELINES**

The ARRIVE guidelines (https://arriveguidelines.org/) are a checklist of recommendations to improve the reporting of research involving animals. Key elements of the study design should be included below to better enable readers to scrutinize the research adequately, evaluate its methodological rigor, and reproduce the methods or findings.

**Study Design**

| **Groups** | **Sex** | **Age** | **Number (prior to experiment)** | **Number (after termination)** | **Littermates (Yes/No)** | **Other description** |
|---|---|---|---|---|---|---|
| Figure 5 | | | | | | |
| *Alk1*$^{f/f}$ *Cdh5 Cre*$^{ERT2}$ *mTmG* | Neonatal mice of both sexes were included without distinction | P8 | 5 | 5 | No | Two retinas from each pup were used for analysis. |
| *Smad4*$^{f/f}$ *Cdh5 Cre*$^{ERT2}$ *mTmG* | | P8 | 4 | 4 | No | |
| *Cdh5 Cre*$^{ERT2}$ *mTmG* | | P8 | 4 | 4 | No | |
| *Alk1*$^{f/f}$ *Cdh5 Cre*$^{ERT2}$ *mTmG* | | P15 | 3 | 3 | No | |
| *Smad4*$^{f/f}$ *Cdh5 Cre*$^{ERT2}$ *mTmG* | | P15 | 7 | 7 | No | |
| *Cdh5 Cre*$^{ERT2}$ *mTmG* | | P15 | 4 | 4 | No | |
| Figure 6 | | | | | | |
| *Alk1*$^{f/f}$ | Neonatal mice of both sexes were included without distinction | P5 | 4 | 4 | Yes | Both retinas from each pup were used for analysis ; however, some were not used due to insufficient quality for reliable quantification |
| *Alk1*$^{f/f}$ *Cdh5 Cre*$^{ERT2}$ | | P5 | 4 | 4 | Yes | |
| *Smad4*$^{f/f}$ | | P5 | 5 | 5 | Yes | |
| *Smad4*$^{f/f}$ *Cdh5 Cre*$^{ERT2}$ | | P5 | 5 | 5 | Yes | |
| *Alk1*$^{f/f}$ | | P6 | 4 | 4 | Yes | |
| *Alk1*$^{f/f}$ *Cdh5 Cre*$^{ERT2}$ | | P6 | 4 | 4 | Yes | |
| *Smad4*$^{f/f}$ | | P6 | 3 | 3 | Yes | |
| *Smad4*$^{f/f}$ *Cdh5 Cre*$^{ERT2}$ | | P6 | 5 | 5 | Yes | |

DOI [to be added]

**Study Design:**

Species, age, sex, strain, and sources of animals are described:

Yes

**Sample Size:**

Exact number of experimental units allocated to each group, the total number in each experiment, the total number of animals used described:

Yes

**Inclusion/exclusion Criteria**

For mosaic experiments several obtained images were not used for analysis due to insufficient quality for reliable quantification.

**Randomization**

Pups of several litters were obtained for each group.

**Statistical Methods**: details of the statistical methods used for each analysis, including software used are described:

Yes

DOI [to be added]
